# The Effect of Rider:Horse Bodyweight Ratio on the Superficial Body Temperature of Horse’s Thoracolumbar Region Evaluated by Advanced Thermal Image Processing

**DOI:** 10.3390/ani12020195

**Published:** 2022-01-13

**Authors:** Małgorzata Domino, Marta Borowska, Anna Trojakowska, Natalia Kozłowska, Łukasz Zdrojkowski, Tomasz Jasiński, Graham Smyth, Małgorzata Maśko

**Affiliations:** 1 Department of Large Animal Diseases and Clinic, Institute of Veterinary Medicine, Warsaw University of Life Sciences (WULS–SGGW), 02-787 Warsaw, Poland; malgorzata_domino@sggw.edu.pl (M.D.); natalia_kozlowska@sggw.edu.pl (N.K.); tomasz_jasinski@sggw.edu.pl (T.J.); 2 Institute of Biomedical Engineering, Faculty of Mechanical Engineering, Białystok University of Technology, 15-351 Bialystok, Poland; m.borowska@pb.edu.pl; 3 The Scientific Society of Veterinary Medicine Students, Warsaw University of Life Sciences, 02-787 Warsaw, Poland; an.trojakowska@gmail.com; 4 Menzies Health Institute Queensland, Griffith University School of Medicine, Southport, QLD 4222, Australia; grahamcsmyth@gmail.com; 5 Department of Animal Breeding, Institute of Animal Science, Warsaw University of Life Sciences (WULS–SGGW), 02-787 Warsaw, Poland

**Keywords:** body mass index, thermograph, texture analysis, color models

## Abstract

**Simple Summary:**

Overloading of the horse’s thoracolumbar region is a serious problem mainly affecting sport and school horses during their daily under-saddle work. As the human population becomes heavier, the effect of rider bodyweight on equine welfare and performance requires further investigation. This study used infrared thermography to assess the effect of rider:horse bodyweight ratio on the horse’s thoracolumbar region by introducing advanced digital image processing. Twelve horses during regular work were ridden by each of six riders assigned to light (L), moderate (M), and heavy (H) groups. Thermal images of the back region were taken before and after standard exercise and underwent conventional analysis and texture analysis where the thermal images were separated into red, green, and blue components. Four areas of the horse’s thoracolumbar region were annotated to represent the withers area, the thoracic spine area, and the left and right areas of back muscles. Among 372 returned features, 75 texture features differed between bodyweight ratio groups, whereas the conventional thermal features did not. Contrary to conventional thermal features, the consistent measurable differences in texture features were evidenced predominantly in the red component of thermal images when the texture heterogeneity measures, such as InvDefMom, SumEntrp, Entropy, DifVarnc, and DifEntrp, were considered.

**Abstract:**

Appropriate matching of rider–horse sizes is becoming an increasingly important issue of riding horses’ care, as the human population becomes heavier. Recently, infrared thermography (IRT) was considered to be effective in differing the effect of 10.6% and 21.3% of the rider:horse bodyweight ratio, but not 10.1% and 15.3%. As IRT images contain many pixels reflecting the complexity of the body’s surface, the pixel relations were assessed by image texture analysis using histogram statistics (HS), gray-level run-length matrix (GLRLM), and gray level co-occurrence matrix (GLCM) approaches. The study aimed to determine differences in texture features of thermal images under the impact of 10–12%, >12 ≤15%, >15 <18% rider:horse bodyweight ratios, respectively. Twelve horses were ridden by each of six riders assigned to light (L), moderate (M), and heavy (H) groups. Thermal images were taken pre- and post-standard exercise and underwent conventional and texture analysis. Texture analysis required image decomposition into red, green, and blue components. Among 372 returned features, 95 HS features, 48 GLRLM features, and 96 GLCH features differed dependent on exercise; whereas 29 HS features, 16 GLRLM features, and 30 GLCH features differed dependent on bodyweight ratio. Contrary to conventional thermal features, the texture heterogeneity measures, InvDefMom, SumEntrp, Entropy, DifVarnc, and DifEntrp, expressed consistent measurable differences when the red component was considered.

## 1. Introduction

Equestrianism is a discipline that involves horses and humans; therefore, their productive interaction is the key to successful, safe, and comfortable horseback riding. As Body Mass Index (BMI) [1,2] relatively increases in the human population, appropriate matching of rider–horse sizes is becoming an increasingly important issue for the care of riding horses. At first glance, rider size does not seem to disturb the riding use of horses. Rider bodyweight is mostly reported to be in the range of 50 to 90 kg in female-dominated equestrianism [3], while horses most commonly lie in the 500–600 kg bodyweight category [4]. Thus, there is a widespread rider:horse bodyweight ratio range between 10 and 20% of horse bodyweight. The maximum permissible load on horseback was estimated for Japanese native horses [5], Arab endurance horses [6], and the Taishuh pony [7] at 29%, 30%, and 43% of horse bodyweight, respectively. One might conclude that the equine potential for carrying heavy loads is enough for horseback riding [5,6] and this misconception is often widespread within equestrian practitioners. Therefore, it is important to make it clear that an inappropriate rider size has been reported to have adverse implications for horse welfare in everyday practice [8].

A comprehensive evaluation of equine welfare and equine quality of life firstly requires evidence of the horse’s subjective experience using behavioral evidences of emotion in the horse [9], and secondly, evaluation of the horse’s body response using physiological indicators [10]. Within physiological indicators, heart rate [11,12,13], heart rate variability [14,15], and cortisol concentration [12,16] are the most commonly measured [10]. However, these measures are at risk of confounding by physical exertion [10,16,17]. Therefore, in the case of horses used for riding, other more specific indicators of equine well-being are required to assess rider:horse interaction. Recently, the infrared thermography (IRT) measurement of the selected regions of the body surface temperature was proposed to assess both the horse’s emotional state [18,19,20] and the horse’s physiological response to an effort [17,21,22,23], also including the evaluation of the impact of the rider:horse bodyweight ratio on the equine organism [24,25].

Increasing the rider:horse bodyweight ratio from 15% to 20%, 25%, and 30% in a submaximal standard exercise test, increased basic physiological parameters and caused post-exercise muscle pain. A significant increase in plasma creatine kinase activity was noted when the rider:horse bodyweight ratio was changed from 25% to 30%. Similarly, heart rates, breathing frequencies, and plasma lactate concentration increased with increasing weight ratio; however, only when starting from a level of 20% rider:horse bodyweight ratio [26]. When the rider:horse bodyweight ratio was increased from 15.3% to 17.2% and then 18.5% by adding weights, no short-term alterations in heart rate, behavior, and gait symmetry were observed [27]. However, an increase in salivary cortisol concentration suggested an increase in response to higher weight [28], as saliva cortisol concentration is a more sensitive indicator of the physical workload than heart rate and heart rate variability [28,29]. Moreover, when horses were ridden by riders of different bodyweights representing 10–12%, >12 ≤15%, >15 <18%, and >20% rider:horse bodyweight ratio, a temporary adverse effect on horse gait and behavior was observed for the heaviest (>20%) and, to a smaller extent, the heavy (>15 <18%) riders in [30]. It was also shown that of two riders representing 10.6% and 21.3% rider:horse bodyweight ratio, the horses ridden by the heavier rider demonstrated increased heart rate and superficial body temperature on the horse’s neck and trunk [24]. However, the effect of riders representing 10.1% and 15.3% rider:horse bodyweight ratio demonstrated no significant difference with respect to five conventional superficial body temperature measurement approaches in contrast with thermal image analysis based on the gray-level matrices (GLM) [25]. As Dyson et al. [30] highlight, following the World Horse Welfare and the British Equestrian Federation, ‘innovative ways should be developed, so that riders can assess if they are the correct weight for their horse’, advanced digital image processing (DIP) has been introduced here to explore the effect of rider:horse bodyweight ratio on thermal images, which may be integrated into everyday equine practice.

IRT produces colorful thermal images, where the color gradient corresponds to the emitted infrared radiation and thus to the distribution of surface body temperatures [21]. Conventional thermal features, such as average, maximal, or minimal temperature, reflect one numerical value each for considered regions of interest (ROIs), whereas IRT images of the body surface, are composed of many pixels that reflect the body’s complex response [22,25,31]. As the pixel relations are assessed by image texture analysis, histogram statistics (HS), gray-level run-length matrix (GLRLM) and gray level co-occurrence matrix (GLCM) approaches have been applied in the detailed evaluation of medical images. The texture operators explore the image by using statistics of pixel distribution to provide complex descriptions of image texture [32,33]. Consequently, texture analyses have successfully been used in advanced diagnostic imaging to improve the richness of information from ultrasound images [34], radiographic images [35,36,37], magnetic resonance images [33,38], and thermal images [25,31,39,40].

The purpose of the study was to determine if any consistent measurable differences exist in texture features of thermal images taken from the equine thoracolumbar region among riders representing different rider:horse bodyweight ratio.

## 2. Materials and Methods

### 2.1. Animals

Twelve Polish warmblood horses (1–12) (six geldings and six mares; mean ± SD: age 9.3 ± 1.8 years, body weight 566.7 ± 13.7 kg, height at the withers 160.3 ± 3.9 cm) participated in the study. All horses were owned by the Warsaw School of Life Sciences (WULS) and were in daily leisure use in the Didactic Stable of Horse Breeding Division. Horses were selected from 19 WULS owned horses according to the inclusion criteria as follows: (i) the absence of clinical signs of disease in basic clinical examination; (ii) the absence of lameness and signs of back pain in a detailed orthopedic examination; (iii) if possible, representing uniform body weight; and (iv) if possible, representing uniform height at the withers. One horse was excluded due to increased respiratory rate and discharge from the nasal cavity, and three horses were each excluded due to increased tension and pain in response to palpation of the thoracolumbar region or low body weight (363.3 ± 15.3 kg) and height (145.3 ± 1.2 cm), respectively. The details of the horses are summarized in Table 1. One week before the start of the study, all horses were examined, measured, and weighed. All horses were evaluated by an experienced DVM of Equine Clinic WULS, Specialist in Diagnostic Imaging (T.J.). A basic clinical examination, including measurement of heart rate, respiratory rate, capillary refill time, and rectal temperature, as well as inspection of mucous membranes and lymph nodes, was conducted according to international veterinary standards [41]. A detailed back examination, including palpation of the thoracolumbar region and evaluation of the presence of tension in the muscles, lumps, abnormal hair wear and reaction to pain, was performed following Martin and Klide’s protocol [41]. A detailed orthopedic examination was performed following guidelines for the lameness evaluation of the athletic horse [42]. All horses were measured at the withers with a standard equine measure (Busse Sportartikel GmbH & Co. KG, Lohne, Germany) and weighed using equine platform weights (Baka-Wag, Bydgoszcz, Poland). The study was approved by the II Local Ethical Committee on Animal Testing in Warsaw on behalf of the National Ethical Committees on Animal Testing (No WAW2/034/2018, day 27 April 2018).

All horses were housed in individual stalls with the same management. Horses were fed with an individually calculated ration of hay, oats, and concentrate according to its nutritional requirements, which was distributed over three feedings per day. A mineral salt block and freshwater were constantly available. All horses were physically fit as general riding school horses taking part in leisure riding for up to 2 h per day, 6 days a week.

One week before the start of the study, all saddles were fitted to each horse following Greve and Dyson’s protocol [43]. Saddles were considered to fit properly after determination of (i) the panels of the saddle, (ii) the type of flocking, and (iii) the balance of the saddle. When the panels were even, uniformly thick, and soft, as well as demonstrating even contact with the horse’s back, that is, the lowest part of both the saddle and horse’s back were aligned and the center of the seat was located horizontally, the saddle was considered to fit. Horse’s saddles were annotated with mean and range 4.3 (4.1–4.5) kg weight, and the rider plus saddle weights were used to calculate a percentage of horse bodyweight. The details of the saddle’s weight are summarized in Table 1.

### 2.2. Riders

Six female riders (A–F) with 5–10 years riding experience and different bodyweights participated in the study. All riders represented similar upper-intermediate training level [44]. All riders were recreational instructors (RI) of the Polish Equestrian Federation (PEF) and currently active (A, C, E, F) or honorary members (B, D) of the Animal Sciences Students Riding Association (ASSRA) in WULS. Rider ages were 24 (A), 42 (B), 27 (C), 35 (D), 24 I, and 31 (F) years, respectively.

On study day one, all riders were measured, and weighed. All riders were measured with a standard equine measure (Busse Sportartikel GmbH & Co. KG, Lohne, Germany) and weighed with the full equestrian equipment using personal weights (Soehnle, Nassau, Germany). Based on the rider’s bodyweight, three groups were determined with mean and range of bodyweight: (i) light (L, 59.0 (58.0–60.0) kg), (ii) moderate (M, 76.0 (77.0–75.0) kg), and (iii) heavy (H, 91.5 (91.0–92.0) kg). The rider’s BMI for the L, M, and H groups was 23.2 (22.9–23.4) kg/m^2^, 27.0 (26.0–27.9) kg/m^2^, and 30.6 (30.8–30.4) kg/m^2^, respectively. Based on BMI, riders were categorized as normal weight (BMI 18.5–24.9 kg/m^2^; rider A and B in L group), overweight (BMI 25.0–29.9 kg/m^2^, rider C and D in M group), and obese (BMI ≥ 30 kg/m^2^, rider E and F in L group) [45].

The rider:horse bodyweight ratio was calculated individually for each rider (A–F) and horse (1–12) combination (R/H) as the ratio of the bodyweight of the horse to the rider plus saddle weight, expressed as a percentage. The rider:horse bodyweight ratios for the L, M, and H groups were 11.2% (10.6–11.8%), 14.2% (13.5–14.9%), and 16.9% (16.3–17.7%), respectively. The current study was designed to pass the criteria of Dyson et al. [30] where the rider:horse bodyweight ratio would be 10–12% (L), >12% ≤15% (M), and >15% <18% (H). Rider details and rider:horse bodyweight ratio were used to annotate thermal images for further evaluation and are summarized in Table 2.

### 2.3. Standardized Exercise Test and Thermal Images Acquisition

All riders and horses of 72 combinations underwent a standardized exercise test used by Dyson et al. [30]. Each horse was to be ridden once by riders L, M, and H. Each horse was to be ridden once every day, and each rider rode two horses every day, thus the measurements were completed over a 6-day test period.

A standardized exercise test was conducted following the protocol described by Dyson et al. [30] in Supplementary Item 2 [30]. A standardized exercise test was performed in a familiar indoor riding arena (20 × 60 m) with constant protection from environmental conditions, such as solar radiation and wind. Ambient temperature (°C) and relative humidity (RH; %) were continuously measured and maintained during the 6-day test period at 20.1 ± 0.9 °C and 50.5 ± 2.8%, respectively. The riding arena was directly connected with the horse’s stable; therefore, horses could participate in the research without having contact with the outside environment. The standardized exercise test included a walk, rising trot, and canter on both sides, and each bout lasted 30 min. The correct diagonal for which the rider sat in trot as well as the correct canter lead were controlled.

All riders and horses of 72 combinations were imaged using IRT twice, pre-exercise and post-exercise, thus 144 thermal images were obtained and further used for DIP. The imaged area of the thoracolumbar region was brushed as to remove dirt and mud 30 min before imaging. The horses were then led to a familiar, enclosed indoor riding arena to acclimatize to imaging conditions. Thermal images were taken using a non-contact thermographic camera (FLIR Therma CAM E25, FLIR Systems Brasil, Sorocaba, Brazil; emissivity (e) 0.99; temperature range between 10.0 and 50.0 °C). The camera was placed at approximately 1.2 m above the imaging area, directly above the L5 dorsal spinous process according to a previously described protocol [25]. After the first IRT imaging, horses were saddled, and a standardized exercise test was conducted. After a standardized exercise test, horses were unsaddled and the second IRT image was obtained. All thermal images were obtained by the same researcher (M.M.).

### 2.4. Digital Image Processing

**Figure 1:**
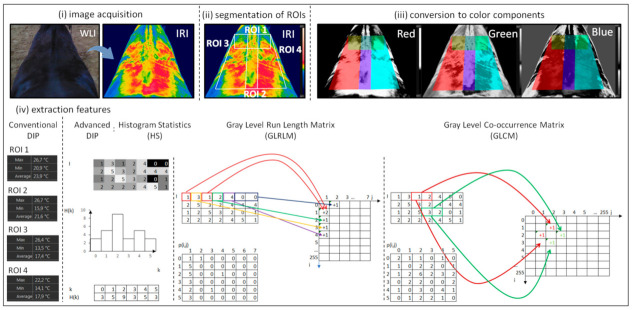
The thermal image processing steps included: (**i**) image acquisition, (**ii**) segmentation of ROIs, (**iii**) conversion to color components, and (**iv**) extraction features for both conventional and advanced digital image processing. WLI—white light; IRT—infrared thermal image; ROI—region of interest; DIP—digital image processing; Min—the minimal temperature; Max—the maximal temperature; Average—the average temperature; HS—histogram statistics and the example of image with its histogram; GLRLM—gray-level run-length matrix with the example of horizontal matrix; GLCM—gray level co-occurrence matrix with the example of matrix for a 1-pixel distance in the horizontal direction.

The thermal image processing steps included: (i) image acquisition, (ii) segmentation of ROIs, (iii) conversion to color components, and (iv) extraction features. Conventional DIP included steps (i), (ii), and (iv), whereas advanced DIP included steps (i–iv). The first two steps (i, ii) were the same for both DIP approaches. The last step (iv) differed between DIPs (Figure 1).

#### 2.4.1. Conventional Digital Image Processing

In the first step (i), the thermal images were acquired according to the protocol described above and saved as .jpg files. The output of the thermal camera is expressed as a thermal image and is also referred to as a thermogram, which is an image color coded for temperature [46]. The thermal images were opened using the software FLIR Tools Professional (FLIR Systems Brasil, Sorocaba, Brazil), and four ROIs were annotated manually as shown in Figure 1. The ROIs represented the withers area (ROI 1), the thoracic spine area (ROI 2), the left area of back musculature (ROI 3), and the right area of back musculature (ROI 4), respectively, and the second step (ii), segmentation of ROIs, has been completed. For the conventional DIP, the third step (iii), conversion to color components, was omitted. Directly after segmentation, the fourth step (iv), features extraction, was conducted as before, using the software FLIR Tools Professional (FLIR Systems Brasil, Sorocaba, Brazil). For each of the four ROIs, the software returned three values of temperatures expressed in ^o^C, which represented three thermal features: the average temperature (Taver), the maximal temperature (Tmax), and the minimal temperature (Tmin).

#### 2.4.2. Advanced Digital Image Processing

In the first (i) and second steps (ii), the thermal images were acquired, saved as .jpg files, and segmented as described above. The thermal images were saved as .bmp files, opened using the QMazda Software [47], and converted to red (R), green (G), and blue (B) components as a way of image transformation to grayscale. As the result of the conversion, each thermal image was represented by three new images, each containing only one color component in grayscale reflecting the two-dimensional temperature assignation of the imaged thoracolumbar region. Afterwards, the third step (iii), conversion to color components, was completed [47,48]. After conversion, the fourth step (iv), the features extraction, was conducted individually for red, green, and blue components as before using the QMazda Software [47,49]. For each of the four ROIs, three analytical approaches: histogram statistics (HS), gray-level run-length matrix (GLRLM), and gray level co-occurrence matrix (GLCM) were applied, thus the QMazda Software returned a total of thirty-one texture features: 13 HS features, 7 GLRLM features, and 11 GLCH features.

In HS, the first order features (FOF) are qualitative measures from the image intensity distribution independent of their location in the image [50]. For image I representing a two-dimensional function, where N and M are the image width and height, respectively, and range of intensity $K\in \left[0..2^{n}-1\right]$ (n is the number of bits per pixel), the normalized histogram H is defined as:

$$H\left(k\right)=\frac{1}{NM}\sum_{i=0}^{N-1}\sum_{j=0}^{M-1}\left\{\begin{array}{c} 1,\;\;\;I\left(i,j\right)=k\\ 0,\;\;\;otherwise \end{array}\right.$$

The histogram H shows the count of pixels in the image that possess a given gray-level value. For most digital images n=8, therefore, the gray-level values range from 0 to 255. Lower values represent darker gray-level (0—black) and higher values represent lighter gray-level (255—white). The example of a digital image with its histogram is shown in Figure 1, and the description was realized by the 13 features calculated from it [50]:

$$FOF_{mean}=\sum_{k=0}^{K}k*H\left(k\right)$$

$$FOF_{variance}=\sum_{k=0}^{K}{\left(k-FOF_{mean}\right)}^{2}*H\left(k\right)$$

$$FOF_{skewness}={\left(FOF_{variance}\right)}^{-3}\sum_{k=0}^{K}{\left(k-FOF_{mean}\right)}^{3}*H\left(k\right)$$

$$FOF_{kurtosis}={\left(FOF_{variance}\right)}^{-4}\sum_{k=0}^{K}{\left(k-FOF_{mean}\right)}^{4}*H\left(k\right)-3$$

$$FOF_{perc01}=\min\left(K\right):\overset{K}{\underset{k=0}{\sum }}H\left(k\right)\geq 0.01$$

$$FOF_{perc10}=\min\left(K\right):\overset{K}{\underset{k=0}{\sum }}H\left(k\right)\geq 0.10$$

$$FOF_{perc50}=\min\left(K\right):\overset{K}{\underset{k=0}{\sum }}H\left(k\right)\geq 0.50$$

$$FOF_{perc90}=\min\left(K\right):\overset{K}{\underset{k=0}{\sum }}H\left(k\right)\geq 0.90$$

$$FOF_{perc99}=\min\left(K\right):\overset{K}{\underset{k=0}{\sum }}H\left(k\right)\geq 0.99$$

$$FOF_{domn01}=k:max\left(\overset{K}{\underset{k=0}{\sum }}H\left(k\right)\right)$$

$$FOF_{domn10}=\;k:max\left(\overset{K-r}{\underset{k=0}{\sum }}\left(H\left(k+r\right)-H\left(k\right)\right)\right)$$

$$FOF_{maxm01}=\frac{1}{NM}max\left(\overset{K}{\underset{k=0}{\sum }}H\left(k\right)\right)$$

$$FOF_{maxm10}=\frac{1}{NM}max\left(\overset{K-r}{\underset{k=0}{\sum }}\left(H\left(k+r\right)-H\left(k\right)\right)\right)$$

Gray-level run-length matrix (GLRLM) features determine the number of runs (the length of similar brightness in one line) of each gray level value in the given direction and for chosen lengths $L=2^{n}-1$ (n—is the number of bits per pixel) [51]. Thus, given a direction (for example, horizontal direction), the GLRLM matrix measures how many times a run of a certain length occur for each gray-level value. The created matrix consists of elements containing information about the brightness and the number of points in one line, for example, $p\left(2,\;1\right)$ indicates a brightness level of 2 and 1 element in the run with such brightness. If there is such a case in the image, the matrix point $p\left(2,1\right)$ is increased by one. The calculation is then performed for successive pixel brightness’s and successive run lengths as is shown in Figure 1. The 7 features from p matrix are defined as [52]:

$$GRLRM_{gray\;level\;non-uniformity}=\frac{1}{n_{p}}\sum_{i=0}^{K}\left(\sum_{j=1}^{L}p\left(i,j\right)\right)$$

$$GRLRM_{run-length\;nonuniformity\;}=\frac{1}{n_{p}}\sum_{j=1}^{L}\left(\sum_{i=0}^{K}p\left(i,j\right)\right)$$

$$GRLRM_{long-run\;emphasis}=\frac{1}{n_{p}}\sum_{i=0}^{K}\sum_{j=1}^{L}j^{2}p\left(i,j\right)$$

$$GRLRM_{short-run\;emphasis}=\frac{1}{n_{p}}\sum_{i=0}^{K}\sum_{j=1}^{L}\frac{p\left(i,j\right)}{j^{2}}$$

$$GRLRM_{fraction\;}=\sum_{i=0}^{K}\sum_{j=1}^{L}\frac{p\left(i,j\right)}{jp\left(i,j\right)}$$

$$GRLRM_{run-length\;nonuniformity\;moment\;}=\frac{1}{n_{p}{}^{2}}\sum_{j=1}^{L}{\left(\sum_{i=0}^{K}p\left(i,j\right)\right)}^{2}$$

$$GRLRM_{gray\;level\;non-uniformity\;moment\;}=\frac{1}{n_{p}{}^{2}}\sum_{i=0}^{K}{\left(\sum_{j=1}^{L}p\left(i,j\right)\right)}^{2}$$

Second order features *(*SOF*)* show the mutual spatial relationship between pairs of image pixels with specific intensity levels. The co-occurrence square matrix (GLCM) p of KxK dimension takes into account the mutual spatial relationship between pairs of image pixels with specific intensity levels $K\;\in \left[0.255\right].$ It is possible to use different distances between pixels pairs and different directions of horizontal, vertical and diagonal neighbors to the matrix calculation. Then, for the selected direction and the selected distance between pixels, the number of pixel pairs that have a given distribution of gray-level values is counted. Thus, each entry in the matrix corresponds to one such distribution of gray levels. For example, let us define a co-occurrence matrix for an 8-bit image with a distance of 1 pixel in the horizontal direction. The size of this matrix will then be 256 × 256. Thus, the element $p\left(1,\;2\right)$ corresponds to the number of pairs of pixels that were found in the image with intensities 1 and 2, respectively. Conversely, the element $p\left(2,\;1\right)$ has exactly the same value and it corresponds to the number of pixel pairs that were found in the image with intensities 2 and 1, respectively. This results, as is shown in Figure 1, in one more element with coordinates of both (1,2) and (2,1). The 11 Haralick features from p matrix are defined as [53]:

$${\mathrm{SOF}}_{\mathrm{AngScMom}}=\sum_{i=1}^{K}\sum_{j=1}^{K}p{\left(i,j\right)}^{2}$$

$${\mathrm{SOF}}_{\mathrm{Contrast}}=\sum_{i=1}^{K}\sum_{j=1}^{K}{\left(i-j\right)}^{2}p\left(i,j\right)$$

$$SOF_{Correlat}=\sum_{i=1}^{K}\sum_{j=1}^{K}\frac{ijp\left(i,j\right)-\mu_{i}\mu_{j}}{\delta_{i}\delta_{j}}$$

$$SOF_{\mathrm{SumOfSqs}}=\sum_{i=1}^{K}\sum_{j=1}^{K}{\left(i-\mu \right)}^{2}p\left(i,j\right)$$

$$SOF_{\mathrm{InvDefMom}}=\sum_{i=1}^{K}\sum_{j=1}^{K}\frac{p\left(i,j\right)}{1+\left|i-j\right|}$$

$$SOF_{\mathrm{SumAverg}}=\sum_{k=2}^{2K}kp_{i+j}\left(k\right)$$

$$SOF_{\mathrm{SumVarnc}}=\sum_{k=2}^{2K}{\left(k-\mu_{i+j}\right)}^{2}p_{i+j}\left(k\right)$$

$$SOF_{\mathrm{SumEntrp}}=-\sum_{k=2}^{2K}p_{i+j}\left(k\right)\log p_{i+j}\left(k\right)$$

$$SOF_{\mathrm{Entropy}}=-\sum_{i=1}^{K}\sum_{j=1}^{K}p\left(i,j\right)\log p\left(i,j\right)$$

$$SOF_{\mathrm{DifVarnc}}=\sum_{k=0}^{K-1}{\left(k-\mu_{i-j}\right)}^{2}p_{i-j}\left(k\right)$$

$$SOF_{\mathrm{DifEntrp}}=-\sum_{k=0}^{K-1}p_{i-j}\left(k\right)\log p_{i-j}\left(k\right)$$

where

$$\mu_{i}=\sum_{i=1}^{K}\sum_{j=1}^{K}ip\left(i,j\right)$$

$$\mu_{j}=\sum_{j=1}^{K}\sum_{j=1}^{K}jp\left(i,j\right)$$

$$\sigma_{i}=\sum_{i=1}^{K}\sum_{j=1}^{K}{\left(i-\mu_{i}\right)}^{2}p\left(i,j\right)$$

$$\sigma_{j}=\sum_{i=1}^{K}\sum_{j=1}^{K}{\left(j-\mu_{j}\right)}^{2}p\left(i,j\right)$$

$$p_{i+j}\left(k\right)=\sum_{i=1}^{K}\sum_{j=1_{i+j=k}}^{K}p\left(i,j\right)$$

$$p_{i-j}\left(k\right)=\sum_{i=1}^{K}\sum_{j=1_{\left|i-j\right|=k}}^{K}p\left(i,j\right)$$

$$\mu_{i+j}=\sum_{k=2}^{2K}kp_{i+j}\left(k\right)$$

$$\mu_{i-j}=\sum_{k=2}^{2K}kp_{i-j}\left(k\right)$$

### 2.5. Data Analysis

Statistical analysis was performed using GraphPad Prism6 software (GraphPad Software Inc., San Diego, CA, USA). Data from 144 images were presented as independent data series of the conventional thermal features (3 IRT features) for the RGB images and the texture features (13 HS features, 7 GLRLM features, and 11 GLCH features) for three image components: red component, green component, and blue component, where each R/H represented one realization. Data series were divided into three groups, L, M, and H, based on the rider and horse bodyweight ratio-dependent criteria, and tested independently for univariate distributions using a Shapiro-Wilk normality test.

The comparisons between the pre-exercise and post-exercise data series were assessed using the Paired t-test for Gaussian data and the Wilcoxon matched-pairs signed-rank test for non-Gaussian data. The alpha value was established as α = 0.05. The numerical data in Supplementary Tables S1–S16 were presented as mean ± standard deviation (SD). Only those features that differed between the pre-exercise and post-exercise imaging for all three groups (L, M, H), simultaneously, were marked by color (light red, green, blue for L group; moderate red, green, blue for M group; and dark red, green, blue for H group) and by a cross (x) in appropriate figure and selected for further analysis.

The comparisons between the L, M, H data series were assessed using the ordinary one-way ANOVA followed by Tukey’s multiple comparisons test for Gaussian data and the Kruskal–Wallis test followed by the Dunn’s multiple comparisons test for non-Gaussian data. The alpha value was established as α = 0.05. Only those features that differed between the L, M, and H groups were marked using color (red, green, blue) and by a cross (x) in appropriate figure. Data on box plots were presented using minimum and maximum values, lower and upper quartiles, and median as well as the mean value were marked by a cross. Additionally, each realization was displayed.

## 3. Results

**Figure 2:**
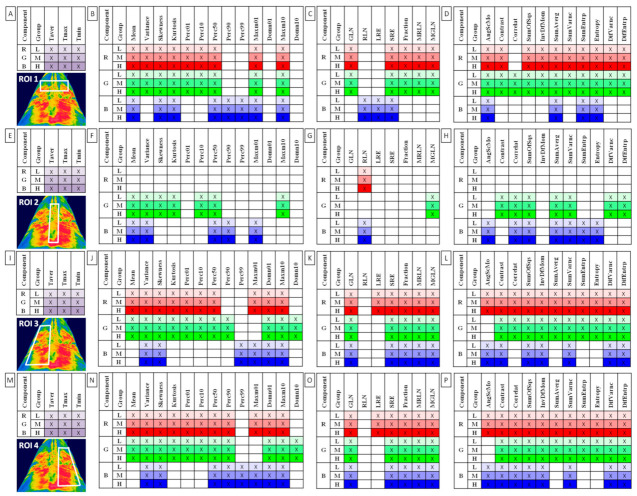
Features of (**A**,**E**,**I**,**M**) conventional thermography, (**B**,**F**,**J**,**N**) histogram statistics, (**C**,**G**,**K**,**Q**) gray-level run-length matrix and (**D**,**H**,**L**,**P**) gray level co-occurrence matrix for examined color components (R, red; G, green, B, blue) were found to be significantly different between the pre-exercise and post-exercise imaging for all rider groups (L, light; M, moderate; H, heavy). Data were presented separately for each region of interest (ROI 1-4) annotated here on the sample thermal image. Taver—average temperature; Tmax—maximal temperature; Tmin—minimal temperature; Skewness—skewness coefficient; Perc01, Perc10, Perc50, Perc90, Perc99—percentiles; Domn01, Domn10—dominants; Maxm01, Maxm10—maximum of moments; GLN—gray level non-uniformity; RLN—run-length nonuniformity; LRE—long-run emphasis; SRE—short-run emphasis; Fraction—the fraction of image in runs; MRLN—run-length nonuniformity moment; MGLN—gray level non-uniformity moment; AngScMom—angular second moment/energy; Correlat—correlation; SumOfSqs—sum of squares; InvDefMom—inverse different moment/homogeneity; SumAverg—summation mean; SumVarnc—summation variance; SumEntrp—summation entropy; DifVarnc—differential variance; DifEntrp—differential entropy.

Among 372 returned combinations of color components (n = 3) and image texture features (n = 31; HS n = 13, GLRLM n = 7, GLCM n = 11) calculated in all ROIs (n = 4), 95 combinations of HS, 48 combinations of GLRLM, and 96 combinations of GLCH differed significantly between the pre-exercise and post-exercise imaging. This is true for all three groups, light (L), moderate (M), and heavy (H). Similarly, all examined conventional thermal features (n = 3) in all ROIs differed significantly between the pre-exercise and post-exercise imaging. These combinations are summarized in Figure 2 and considered for further analysis. The details of this initial comparison as well as the values (mean ± SD) of all examined features were presented in Supplementary Tables S1–S16 available online.

One can observe that all conventional thermal features differed after exercise in all examined ROIs, whereas texture features differed in ROI 1, ROI 3, and ROI 4 rather than in ROI 2. Moreover, a high degree of symmetry was observed between the left and right area of back musculature, thus only Perc90 of the red component as well as Perc90, Perc99, and Correlat of the blue component were significantly different between ROIs 3 and 4.

**Figure 3:**
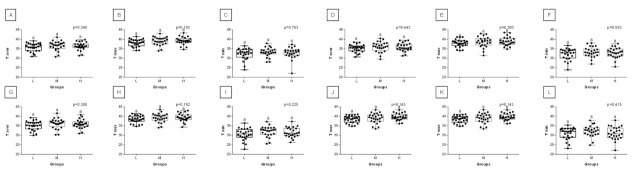
Features of conventional thermography compared between the light (L), moderate (M), and heavy (H) groups in consecutive regions of interest (ROI 1, (**A**–**C**); ROI 2, (**D**–**F**); ROI 3, (**G**–**I**); ROI 4, (**J**–**L**)). Differences between groups were indicated with individual *p*-values when *p* < 0.05. Different superscripts on each plot were statistically different. (**A**,**D**,**G**,**J**) Taver—average temperature; (**B**,**E**,**H**,**K**) Tmax—maximal temperature; (**C**,**F**,**I**,**L**) Tmin—minimal temperature.

**Figure 4:**
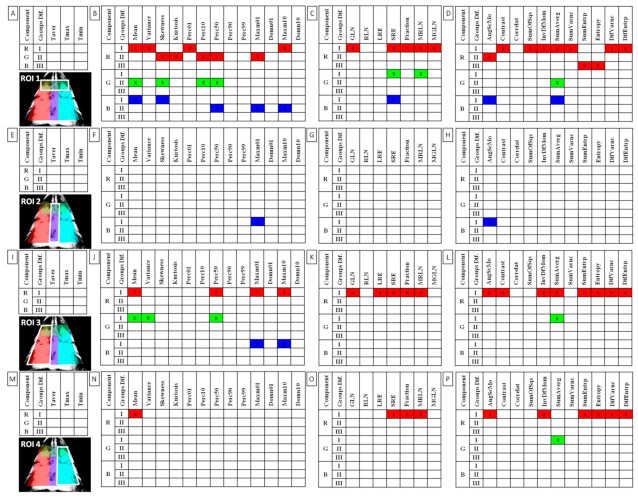
Features of (**A**,**E**,**I**,**M**) conventional thermography, (**B**,**F**,**J**,**N**) histogram statistics, (**C**,**G**,**K**,**Q**) gray-level run-length matrix and (**D**,**H**,**L**,**P**) gray level co-occurrence matrix for examined color components (R, red; G, green, B, blue) found to be significantly different between the light (L), moderate (M), and heavy (H) groups. Three groups of differences (Groups Dif.) were separated: I—groups L and H differed (marked as a, ab, b on plots); II—groups L/M and H differed (marked as a, a, b on plots); III—groups L, M, H differed (marked as a, b, c on plots). Data were presented separately for each region of interest (ROI 1–4) annotated here on the sample thermal image of the red component. Taver—average temperature; Tmax—maximal temperature; Tmin—minimal temperature; Skewness—skewness coefficient; Perc01, Perc10, Perc50, Perc90, Perc99—percentiles; Domn01, Domn10—dominants; Maxm01, Maxm10—maximum of moments; GLN—gray level non-uniformity; RLN—run-length nonuniformity; LRE—long-run emphasis; SRE—short-run emphasis; Fraction—a fraction of image in runs; MRLN—run-length nonuniformity moment; MGLN—gray level non-uniformity moment; AngScMom—angular second moment/energy; Correlat—correlation; SumOfSqs—sum of squares; InvDefMom—inverse different moment/homogeneity; SumAverg—summation mean; SumVarnc—summation variance; SumEntrp—summation entropy; DifVarnc—differential variance; DifEntrp—differential entropy.

**Figure 5:**
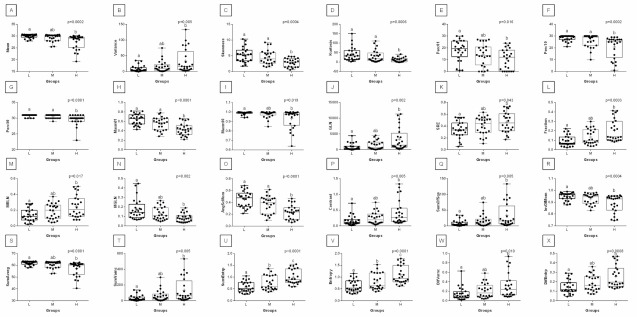
Analysis of texture features for the red component compared between light (L), moderate (M), and heavy (H) groups in the first region of interest (ROI 1). Differences between groups were indicated with individual *p*-values when *p* < 0.05. Different superscripts on each plot were statistically different. (**A**) Mean; (**B**) Variance; (**C**) Skewness—skewness coefficient; (**D**) Kurtosis; (**E**–**G**) Perc01, Perc10, Perc50—percentiles; (**H**,**I**) Maxm01, Maxm10—maximum of moments; (**J**) GLN—gray level non-uniformity; (**K**) SRE—short-run emphasis; (**L**) Fraction—a fraction of image in runs; (**M**) MRLN—run-length nonuniformity moment; (**N**) MGLN—gray level non-uniformity moment; (**O**) AngScMom—angular second moment/energy; (**P**) Contrast; (**Q**) SumOfSqs—sum of squares; (**R**) InvDefMom—inverse different moment/homogeneity; (**S**) SumAverg—summation mean; (**T**) SumVarnc—summation variance; (**U**) SumEntrp—summation entropy; (**V**) Entropy; (**W**) DifVarnc—differential variance; (**X**) DifEntrp—differential entropy. Data are presented using minimum and maximum values, lower and upper quartiles, and median. The mean value is marked by a cross.

**Figure 6:**
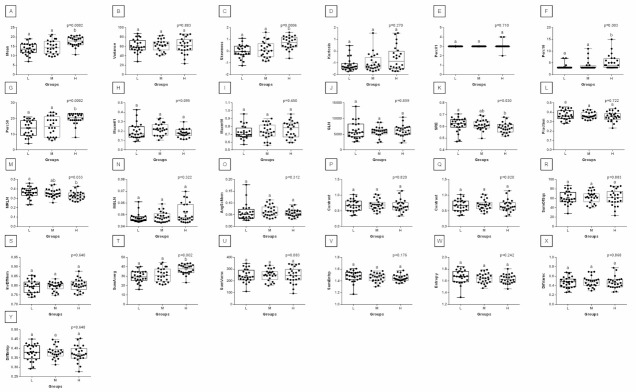
Analysis of texture features for the green component compared between light (L), moderate (M), and heavy (H) groups in the first region of interest (ROI 1). Differences between groups were indicated with individual *p*-values when *p* < 0.05. Different superscripts on each plot were statistically different. (**A**) Mean; (**B**) Variance; (**C**) Skewness—skewness coefficient; (**D**) Kurtosis; (**E**–**G**) Perc01, Perc10, Perc50—percentiles; (**H**,**I**) Maxm01, Maxm10—maximum of moments; (**J**) GLN—gray level non-uniformity; (**K**) SRE—short-run emphasis; (**L**) Fraction—a fraction of image in runs; (**M**) MRLN—run-length nonuniformity moment; (**N**) MGLN—gray level non-uniformity moment; (**O**) AngScMom—angular second moment/energy; (**P**) Contrast; (**Q**) Correlate; (**R**) SumOfSqs—sum of squares; (**S**) InvDefMom—inverse different moment/homogeneity; (**T**) SumAverg—summation mean; (**U**) SumVarnc—summation variance; (**V**) SumEntrp—summation entropy; (**W**) Entropy; (**X**) DifVarnc—differential variance; (**Y**) DifEntrp—differential entropy. Data are presented using minimum and maximum values, lower and upper quartiles, and median. The mean value is marked by a cross.

**Figure 7:**
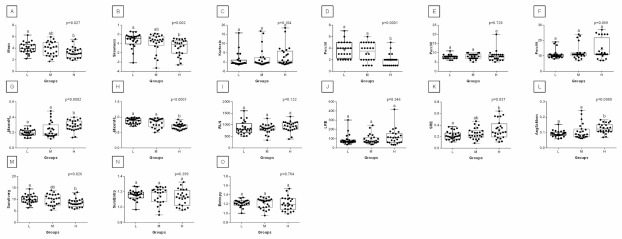
Analysis of texture features for the blue component compared between light (L), moderate (M), and heavy (H) groups in the first regions of interest (ROI 1). Differences between groups were indicated with individual *p*-values when *p* < 0.05. Different superscripts on each plot were statistically different. (**A**) Mean; (**B**) Skewness—skewness coefficient; (**C**) Kurtosis; (**D**–**F**) Perc50, Perc90, Perc99—percentiles; (**G**,**H**) Maxm01, Maxm10—maximum of moments; (**I**) RLN—run-length nonuniformity; (**J**) LRE—long-run emphasis; (**K**) SRE—short-run emphasis; (**L**) AngScMom—angular second moment/energy; (**M**) SumAverg—summation mean; (**N**) SumEntrp—summation entropy; (**O**) Entropy. Data are presented using minimum and maximum values, lower and upper quartiles, and median. The mean value is marked by a cross.

**Figure 8:**
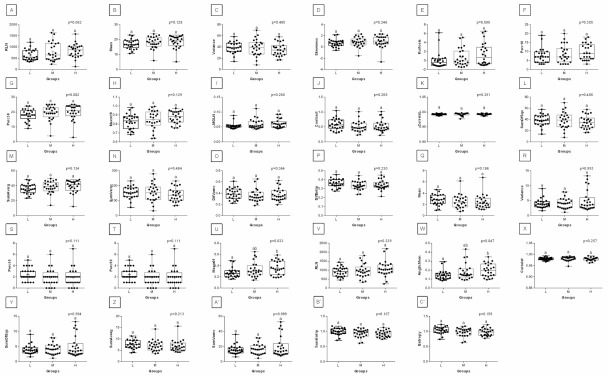
Analysis of texture features for the red, green, and blue components compared between light (L), moderate (M), and heavy (H) groups in the second region of interest (ROI 2). Differences between groups were indicated with individual *p*-values when *p* < 0.05. Different superscripts on each plot were statistically different. For the red component: (**A**) RLN—run-length nonuniformity. For the green component: (**B**) Mean; (**C**) Variance; (**D**) Skewness—skewness coefficient; (**E**) Kurtosis; (**F**,**G**) Perc10, Perc50—percentiles; (**H**) Maxm10—maximum of moments; (**I**) MGLN—gray level non-uniformity moment; (**J**) Contrast; (**K**) Correlat—correlation; (**L**) SumOfSqs—sum of squares; (**M**) SumAverg—summation mean; (**N**) SumVarnc—summation variance; (**O**) DifVarnc—differential variance; (**P**) DifEntrp—differential entropy. For the blue component: (**Q**) Mean; (**R**) Variance; (**S**,**T**) Perc50, Perc90—percentiles; (**U**) Maxm01—maximum of moments; (**V**) RLN—run-length nonuniformity; (**W**) AngScMom—angular second moment/energy; (**X**) Correlat—correlation; (**Y**) SumOfSqs—sum of squares; (**Z**) SumAverg—summation mean; (**A’**) SumVarnc—summation variance; (**B’**) SumEntrp—summation entropy; (**C’**) Entropy. Data are presented using minimum and maximum values, lower and upper quartiles, and median. The mean value is marked by a cross.

**Figure 9:**
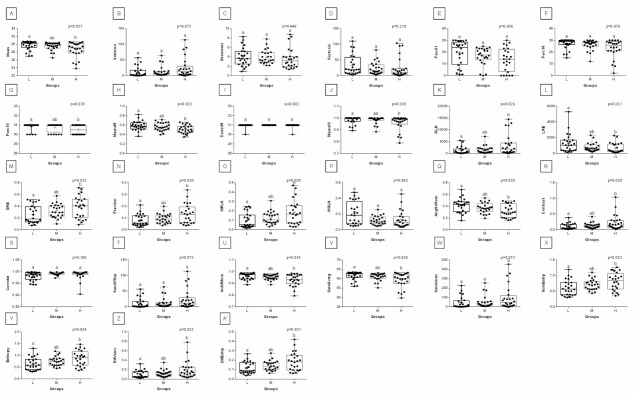
Analysis of texture features for the red component compared between light (L), moderate (M), and heavy (H) groups in the third region of interest (ROI 3). Differences between groups were indicated with individual *p*-values when *p* < 0.05. Different superscripts on each plot were statistically different. (**A**) Mean; (**B**) Variance; (**C**) Skewness—skewness coefficient; (**D**) Kurtosis; (**E**–**G**) Perc01, Perc10, Perc50—percentiles; (**H**,**J**) Maxm01, Maxm10—maximum of moments; (**I**) Domn01—dominant; (**K**) GLN—gray level non-uniformity; (**L**) LRE—long-run emphasis; (**M**) SRE—short-run emphasis; (**N**) Fraction—a fraction of image in runs; (**O**) MRLN—run-length nonuniformity moment; (**P**) MGLN—gray level non-uniformity moment; (**Q**) AngScMom—angular second moment/energy; (**R**) Contrast; (**S**) Correlat—correlation; (**T**) SumOfSqs—sum of squares; (**U**) InvDefMom—inverse different moment/homogeneity; (**V**) SumAverg—summation mean; (**W**) SumVarnc—summation variance; (**X**) SumEntrp—summation entropy; (**Y**) Entropy; (**Z**) DifVarnc—differential variance; (**A’**) DifEntrp—differential entropy. Data are presented using minimum and maximum values, lower and upper quartiles, and median. The mean value is marked by a cross.

**Figure 10:**
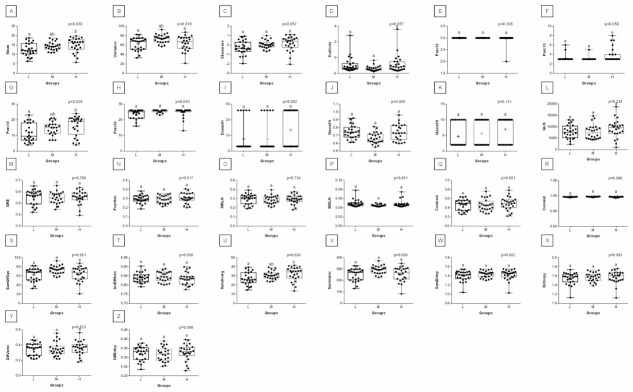
Analysis of texture features for the green component compared between light (L), moderate (M), and heavy (H) groups in the third region of interest (ROI 3). Differences between groups were indicated with individual *p*-values when *p* < 0.05. Different superscripts on each plot were statistically different. (**A**) Mean; (**B**) Variance; (**C**) Skewness—skewness coefficient; (**D**) Kurtosis; (**E**–**H**) Perc01, Perc10, Perc50, Perc90—percentiles; (**I**,**K**) Maxm01, Maxm10—maximum of moments; (**J**) Domn01—dominant; (**L**) GLN—gray level non-uniformity; (**M**) SRE—short-run emphasis; (**N**) Fraction—a fraction of image in runs; (**O**) MRLN—run-length nonuniformity moment; (**P**) MGLN—gray level non-uniformity moment; (**Q**) Contrast; (**R**) Correlat—correlation; (**S**) SumOfSqs—sum of squares; (**T**) InvDefMom—inverse different moment/homogeneity; (**U**) SumAverg—summation mean; (**V**) SumVarnc—summation variance; (**W**) SumEntrp—summation entropy; (**X**) Entropy; (**Y**) DifVarnc—differential variance; (**Z**) DifEntrp—differential entropy. Data are presented using minimum and maximum values, lower and upper quartiles, and median. The mean value is marked by a cross.

**Figure 11:**
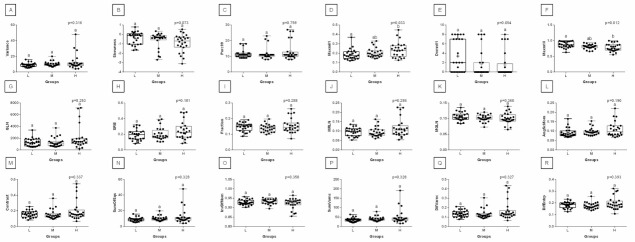
Analysis of texture features for the blue component compared between light (L), moderate (M), and heavy (H) groups in the third region of interest (ROI 3). Differences between groups were indicated with individual *p*-values when *p* < 0.05. Different superscripts on each plot were statistically different. (**A**) Variance; (**B**) Skewness—skewness coefficient; (**C**) Perc99—percentile; (**D**,**F**) Maxm01, Maxm10—maximum of moments; (**E**) Domn01—dominant; (**G**) GLN—gray level non-uniformity; (**H**) SRE—short-run emphasis; (**I**) Fraction—a fraction of image in runs; (**J**) MRLN—run-length nonuniformity moment; (**K**) MGLN—gray level non-uniformity moment; (**L**) AngScMom—angular second moment/energy; (**M**) Contrast; (**N**) SumOfSqs—sum of squares; (**O**) InvDefMom—inverse different moment/homogeneity; (**P**) SumVarnc—summation variance; (**Q**) DifVarnc—differential variance; (**R**) DifEntrp—differential entropy. Data are presented using minimum and maximum values, lower and upper quartiles, and median. The mean value is marked by a cross.

**Figure 12:**
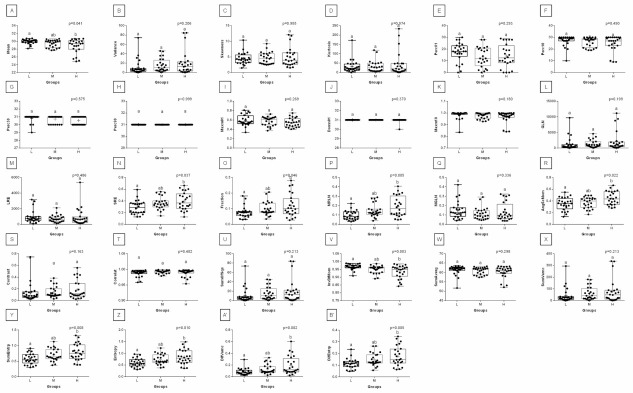
Analysis of texture features for the red component compared between light (L), moderate (M), and heavy (H) groups in the fourth region of interest (ROI 4). Differences between groups were indicated with individual *p*-values when *p* < 0.05. Different superscripts on each plot were statistically different. (**A**) Mean; (**B**) Variance; (**C**) Skewness—skewness coefficient; (**D**) Kurtosis; (**E**–**H**) Perc01, Perc10, Perc50, Perc90—percentiles; (**I**,**K**) Maxm01, Maxm10—maximum of moments; (**J**) Domn01—dominant; (**L**) GLN—gray level non-uniformity; (**M**) LRE—long-run emphasis; (**N**) SRE—short-run emphasis; (**O**) Fraction—a fraction of image in runs; (**P**) MRLN—run-length nonuniformity moment; (**Q**) MGLN—gray level non-uniformity moment; (**R**) AngScMom—angular second moment/energy; (**S**) Contrast; (**T**) Correlat—correlation; (**U**) SumOfSqs—sum of squares; (**V**) InvDefMom—inverse different moment/homogeneity; (**W**) SumAverg—summation mean; (**X**) SumVarnc—summation variance; (**Y**) SumEntrp—summation entropy; (**Z**) Entropy; (**A’**) DifVarnc—differential variance; (**B’**) DifEntrp—differential entropy. Data are presented using minimum and maximum values, lower and upper quartiles, and median. The mean value is marked by a cross.

**Figure 13:**
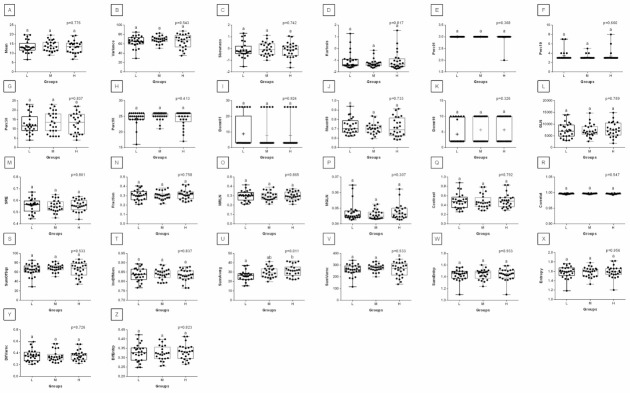
Analysis of texture features for the green component compared between light (L), moderate (M), and heavy (H) groups in the fourth region of interest (ROI 4). Differences between groups were indicated with individual *p*-values when *p* < 0.05. Different superscripts on each plot were statistically different. (**A**) Mean; (**B**) Variance; (**C**) Skewness—skewness coefficient; (**D**) Kurtosis; (**E**–**H**) Perc01, Perc10, Perc50, Perc90—percentiles; (**I**,**K**) Maxm01, Maxm10—maximum of moments; (**J**) Domn01—dominant; (**L**) GLN—gray level non-uniformity; (**M**) SRE—short-run emphasis; (**N**) Fraction—a fraction of image in runs; (**O**) MRLN—run-length nonuniformity moment; (**P**) MGLN—gray level non-uniformity moment; (**Q**) Contrast; (**R**) Correlat—correlation; (**S**) SumOfSqs—sum of squares; (**T**) InvDefMom—inverse different moment/homogeneity; (**U**) SumAverg—summation mean; (**V**) SumVarnc—summation variance; (**W**) SumEntrp—summation entropy; (**X**) Entropy; (**Y**) DifVarnc—differential variance; (**Z**) DifEntrp—differential entropy. Data are presented using minimum and maximum values, lower and upper quartiles, and median. The mean value is marked by a cross.

**Figure 14:**
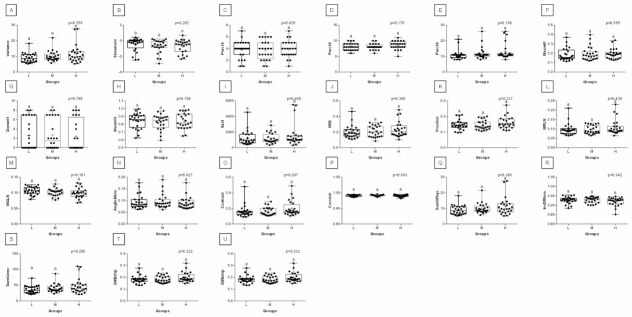
Analysis of texture features for the blue component compared between light (L), moderate (M), and heavy (H) groups in the fourth region of interest (ROI 4). Differences between groups were indicated with individual *p*-values when *p* < 0.05. Different superscripts on each plot were statistically different. (**A**) Variance; (**B**) Skewness—skewness coefficient; (**C**–**E**) Perc50, Perc90, Perc99—percentiles; (**F**,**H**) Maxm01, Maxm10—maximum of moments; (**G**) Domn01—dominant; (**I**) GLN—gray level non-uniformity; (**J**) SRE—short-run emphasis; (**K**) Fraction—a fraction of image in runs; (**L**) MRLN—run-length nonuniformity moment; (**M**) MGLN—gray level non-uniformity moment; (**N**) AngScMom—angular second moment/energy; (**O**) Contrast; (**P**) Correlat—correlation; (**Q**) SumOfSqs—sum of squares; (**R**) InvDefMom—inverse different moment/homogeneity; (**S**) SumVarnc—summation variance; (**T**) DifVarnc—differential variance; (**U**) DifEntrp—differential entropy. Data are presented using minimum and maximum values, lower and upper quartiles, and median. The mean value is marked by a cross.

When comparing post-exercise conventional thermal features between rider groups, no difference was found in ROI 1, ROI 2, ROI 3, and ROI 4 (Figure 3). However, when the post-exercise texture features were similarly compared, among 239 examined combinations, rider:horse bodyweight ratio-dependent differences were observed for 29 combinations of HS, 16 combinations of GLRLM, and 30 combinations of GLCH. These combinations were summarized in Figure 4, whereas the detailed comparisons were presented in Figure 5, Figure 6, Figure 7, Figure 8, Figure 9, Figure 10, Figure 11, Figure 12, Figure 13 and Figure 14.

One can observe that among texture features, most of the rider:horse bodyweight ratio-dependent differences are seen in ROI 1. Considering the red component in ROI 1, groups L and H differed in Mean, Variance, Perc01, Maxm10, GLN, SRE, Fraction, MRLN, MGLN, Contrast, SumOfSqs, InvDifMom, SumAverg, SumVarnc, DifVarnc, and DifEntp whereas, both groups L and M differed from group H in Skewness, Kurtosis, Perc10, Perc50, Maxm01, and AngScMo. Moreover, the values of two GLCM features, SumEntrp and Entropy, were different in groups L, M, and H (Figure 5). Considering the green component in ROI 1, groups L and H differed in SRE and MRLN whereas, both groups L and M differed from group H in Mean, Skewness, Perc10, Perc50, and SumAverg (Figure 6). Considering the blue component in ROI 1, groups L and H differed in Mean, Skewness, SRE, and SumAverg whereas, both groups L and M differed from group H for Perc50, Maxm01, Maxm10, and AngScMo (Figure 7).

Contrary to ROI 1, the fewest rider:horse bodyweight ratio-dependent texture features were observed in ROI 2, therefore comparisons of the red, green, and blue components were displayed on one plot. In ROI 2, only two features, Maxm01, and AngScMo were different in groups L and H (Figure 8).

With respect to ROIs 3 and 4, a much lower degree of symmetry was observed between the left and right area of back muscles. Only Mean, SRE, Fraction, MRLN, AngScMom, InvDfMom, SumEntrp, Entropy, DifVarnc and DifEntrp of the red component as well as SumAverg of the blue component differed between groups L and H, for ROIs 3 and 4 (Figure 9, Figure 10, Figure 11, Figure 12, Figure 13 and Figure 14). Furthermore, considering the red component in ROI 3, groups L and H differed in Perc50, Maxm01, Maxm10, GLN, LRE, Contrast, and SumAverg (Figure 9). Considering the green component in ROI 3, groups L and H differed in Mean, Variance, and Perc50 (Figure 10). Finally, considering the blue component in ROI 3, groups L and H differed in Maxm01 and Maxm10 (Figure 11). For all other examined features, no rider:horse bodyweight ratio-dependent differences were found.

## 4. Discussion

The evaluation of the texture of IRT images makes it possible to identify the rider:horse bodyweight ratio-related alterations to a horse’s organism’s physiology during standardized exercise test conditions. The assessment of the correct weight of riders for their horse is a multifactorial issue with many inter-related aspects [8]. Therefore, it is not surprising that conventional thermal image features did not elucidate the effect of rider:horse bodyweight ratio, which was in an acceptable range of 10–20% and the horse’s thoracolumbar region was not overloaded >20% [26,30]. Our results do not contradict the literature that states that overload (rider:horse bodyweight ratio 21.3%) may be reflected by an increase in heart rate and superficial body temperature of the horse’s neck and trunk [24], as we did not include this higher rider:horse bodyweight ratio group. Our results support the recent findings that the effect of rider:horse bodyweight ratio (10–20%) cannot be distinguished with conventional thermal features [25].

Wilk et al. [24] observed that the average superficial body temperature on some, but not all, body parts of exercised horses increased more when horses were worked under a load of >20% than under a load of 10% of their bodyweight. This early research on the application of IRT indicated that the neck and trunk regions are the most suitable for determination of the thermal effects of rider:horse bodyweight ratio. Therefore, our preliminary [25] and current studies were focused on the thoracolumbar region. Moreover, being aware of the limitations of IRT, including the influence of ambient temperature [54], the warming effect of sunlight exposure [21], and the cooling effect of air flow during movement [55], the under-saddle region seems to be the least susceptible to the influence of external factors and best reflects the thermal dynamics in the location of direct interaction between the rider and the horse. The current research was designed to extend the recent application of IRT imaging-based identification of the rider:horse bodyweight ratio-related alterations, by including several riders in the same weight category [24], evaluating a larger region of the trunk [25], and significantly broadening the DIP protocol by using three color components [56,57], and three texture analysis approaches [50,51,52,53].

In our previous research, GLN, RLN, LRE, SRE, Fraction, MRLN, Contras, Correlate, InvDefMom, Entropy, DifVarnc, and DifEntrp differed with exercise and with rider:horse bodyweight ratio in the 10.1% and 15.3% bodyweight ratio groups [25]. In the current study, when three bodyweight ratio groups were investigated, 11.2% (10.6–11.8%), 14.2% (13.5–14.9%), and 16.9% (16.3–17.7%), the same GLRLM and GLCM features differed between pre-exercise and post-exercise images. However, after decomposing the thermal image into color components and examining more ROIs, GLN, LRE, SRE, Fraction, MRLN, and MGLN have been shown to better reflect the texture of muscle-rich areas (ROI 1, 3, and 4), whereas RLN better reflects the texture of muscle-poor areas (ROI 2). Moreover, in the muscle-poor area the participation of the features for the red component in the general pattern was poor, whereas in the muscle-rich areas, the participation of the features for the red component was predominant. In thermal images, high temperature is red annotated and low temperature is blue annotated [21]. Therefore, red component domination in ROIs 1, 3, and 4, and lack thereof in ROI 2 is supported by the recent findings describing effort-dependent [58] and muscle-size dependent [23,59] increases in heat emission from the body surface in horses. It is worth noting that in the red component, SRE, Fraction, and MRLN from the GLRLM approach and InvDefMom, SumEntrp, Entropy, DifVarnc, and DifEntrp differed depending on the rider:horse bodyweight ratio in all three muscle-rich areas. Moreover, only the SumEntrp and Entropy for the red component in ROI 1, differed between all three bodyweight ratio groups, 11.2%, 14.2%, and 16.9%. These entropy-related features have been suggested to reflect the increased degree of thermal energy dissipation [25,31,40], indicating a high heterogeneity of texture [26,60].

Dyson et al. [30] demonstrated major and minor gait asymmetries when horses were worked under a load of >20% and >15%<18% of their bodyweight, respectively, and suggested that there were biomechanical effects on gait asymmetry caused by a change in load on the horse’s back. As some signs of the asymmetry in texture of thermal images were reported here, one might expect that texture analysis may be helpful in the assessment of the correct rider weight for their horse. Conventional IRT features did not differ between loads. However, further research is required with simultaneous IMUs gait assessment [30] and advanced DIP of thermal images obtained from the thoracolumbar [25], segmented trunk, and neck [24] regions of the horse. Our results support the recent findings [25] and suggest that the red component of IRT images may be useful in subsequent, more application-based research on the utilization of advanced DIP of IRT as a tool to improve the comfort of riding horses.

Dyson et al. [30] investigated inertial measurement units (IMUs), a force back-load-mat, subjective gait assessment, and 24 behavioral markers that are difficult to use in daily practice. Wilk et al. [24] introduced IRT for simple demonstration of differing load influence on the horse’s body. Regardless of the measurement method used, however, it is agreed that knowingly assessing the rider:horse bodyweight ratio will improve horse welfare as in many riding schools and equestrian disciplines both in UK [30] and Poland [61], a horse and pony are ridden by riders of varying BMI. Such assessment, following Dyson et al. [30], should be useful in the everyday equine practice, where a horse performs the under-rider overground exercise with turns and circles. As the practical application of modern technologies is developing dynamically both in the field of equine thermal imaging [62,63,64] and DIP of medical images [31,33,36,37,38,39,40], each new approach can ultimately reach every horse owner.

We believe the assessment of IRT image texture heterogeneity in the red component may be easily and accurately transferred into typical daily riding practice since the smartphone thermal camera software and medical application are developing rapidly [62,65,66,67]. In this way, the currently described science advancement in non-invasive IRT imaging and advanced DIP will be leveraged to improve the practical assessment of the appropriate matching of rider:horse sizes. The direct information, if the horse’s thoracolumbar region is not overloaded and a particular rider is not too heavy for that horse, is necessary to assess the impact of riding and identify factors that require changes. We support the belief that all horse owners and carers should be able to assess the horses:human interaction and thus improve the quality of life of horses under their care [9]. Therefore, we presented here the first step of successive development of technology, which in the long run may improve welfare outcomes for the horses used in equestrian activities. It is worth noting that current advancement allows detecting much more subtle changes of physiological measures of a horse’s thoracolumbar region in response to the horses:human interaction during riding work than has been recently available [21,24,25]. However, it should be kept in mind that physiological measures without taking into account the behavioral evidence of a horse’s emotion have limited use when assessing horse welfare [9,10]. Therefore, the main limitation of this study is disregarding the evaluation of horse’s emotions and horse’s experiences and therefore should be considered as preliminary. In further research, the rider position in the saddle, as a function of rider size and skill [30], the effect of inappropriate saddle fit [42,64,68], the impact of different environmental conditions [21,54,55], and most importantly an evaluation of the equine behavior indicative of underlying mood state and general well-being of horses [9,15,16] should be considered to explain the role of this horses:human interaction assessment in the overall improvement of the equine quality of life.

## 5. Conclusions

Based on the presented results, one may conclude that consistent measurable differences exist in texture features between thermal images taken from the thoracolumbar region of horses worked under riders with a different rider:horse bodyweight ratio. The differences were observed primarily in the red component of IRT images and especially in texture heterogeneity measures, such as InvDefMom, SumEntrp, Entropy, DifVarnc, and DifEntrp. The extension of the conventional DIP of IRT images by advanced approaches proposed here, makes it possible to identify the rider bodyweight-related alterations to a horse’s thoracolumbar region loaded in the range of 10–12%, >12 ≤15%, >15 <18% rider:horse bodyweight ratios.

## Figures and Tables

**Table 1:** Details of six riders (A–F), representing light (L), moderate (M), and heavy (H) groups, and twelve horses (1–12) that participated in the study.

		Rider				Horse				Horse		
Group	Sign	BW	High	BMI	Sign	BW	High	SW	Sign	BW	High	SW
L	A	58	159	22.9	1	585	166	4.1	7	560	162	4.2
	B	60	160	23.4	2	550	154	4.5	8	545	157	4.2
M	C	77	172	26.0	3	580	158	4.2	9	570	158	4.4
	D	75	164	27.9	4	575	164	4.5	10	580	162	4.4
H	E	91	172	30.8	5	560	160	4.1	11	565	160	4.2
	F	92	174	30.4	6	550	156	4.4	12	580	166	4.2

BW—bodyweight (kg); height (cm); BMI—body mass index (kg/m^2^); SW—saddle’s weight (kg).

**Table 2:** The rider:horse bodyweight ratio calculated for combinations (R/H) of six riders (A–F), representing light (L), moderate (M), and heavy (H) groups, and twelve horses (1–12).

	Rider: Horse Bodyweight Ratio												
Group	R/H	1	2	3	4	5	6	7	8	9	10	11	12
L	A	10.6	11.4	10.7	10.9	11.1	11.3	11.1	11.4	10.9	10.8	11.0	10.7
	B	11.0	11.7	11.1	11.2	11.4	11.7	11.5	11.8	11.3	11.1	11.4	11.1
M	C	13.9	14.8	14.0	14.2	14.5	14.8	14.5	14.9	14.3	14.0	14.4	14.0
	D	13.5	14.5	13.7	13.8	14.1	14.4	14.1	14.5	13.9	13.7	14.0	13.7
H	E	16.3	17.4	16.4	16.6	17.0	17.3	17.0	17.5	16.7	16.4	16.8	16.4
	F	16.4	17.5	16.6	16.8	17.2	17.5	17.2	17.7	16.9	16.6	17.0	16.6

## Data Availability

The data presented in this study are available on request from the corresponding author.

## Supplementary Materials

The following are available online at https://www.mdpi.com/article/10.3390/ani12020195/s1, Table S1: The withers area (ROI 1). Values (mean ± SD) of conventional thermal features in light (L), moderate (M), and heavy (H) groups. The pre-exercise (pre-ex) and post-exercise (post-ex) data series were compared; Table S2: The thoracic spine area (ROI 2). Values (mean ± SD) of conventional thermal features in light (L), moderate (M), and heavy (H) groups. The pre-exercise (pre-ex) and post-exercise (post-ex) data series were compared; Table S3: The left area of back musculature (ROI 3). Values (mean ± SD) of conventional thermal features in light (L), moderate (M), and heavy (H) groups. The pre-exercise (pre-ex) and post-exercise (post-ex) data series were compared; Table S4: The right area of back musculature (ROI 4). Values (mean ± SD) of conventional thermal features in light (L), moderate (M), and heavy (H) groups. The pre-exercise (pre-ex) and post-exercise (post-ex) data series were compared; Table S5: The withers area (ROI 1). Values (mean ± SD) of histogram statistics (HS) features for three color components (R, red; G, green; B, blue) in light (L), moderate (M), and heavy (H) groups. The pre-exercise (pre-ex) and post-exercise (post-ex) data series were compared; Table S6: The thoracic spine area (ROI 2). Values (mean ± SD) of histogram statistics (HS) features for three color components (R, red; G, green; B, blue) in light (L), moderate (M), and heavy (H) groups. The pre-exercise (pre-ex) and post-exercise (post-ex) data series were compared; Table S7: The left area of back musculature (ROI 3). Values (mean ± SD) of histogram statistics (HS) features for three color components (R, red; G, green; B, blue) in light (L), moderate (M), and heavy (H) groups. The pre-exercise (pre-ex) and post-exercise (post-ex) data series were compared; Table S8: The right area of back musculature (ROI 4). Values (mean ± SD) of histogram statistics (HS) features for three color components (R, red; G, green; B, blue) in light (L), moderate (M), and heavy (H) groups. The pre-exercise (pre-ex) and post-exercise (post-ex) data series were compared; Table S9: The withers area (ROI 1). Values (mean ± SD) of gray-level run-length matrix (GLRLM) features for three color components (R, red; G, green; B, blue) in light (L), moderate (M), and heavy (H) groups. The pre-exercise (pre-ex) and post-exercise (post-ex) data series were compared; Table S10: The thoracic spine area (ROI 2). Values (mean ± SD) of gray-level run-length matrix (GLRLM) features for three color components (R, red; G, green; B, blue) in light (L), moderate (M), and heavy (H) groups. The pre-exercise (pre-ex) and post-exercise (post-ex) data series were compared; Table S11: The left area of back musculature (ROI 3). Values (mean ± SD) of gray-level run-length matrix (GLRLM) features for three color components (R, red; G, green; B, blue) in light (L), moderate (M), and heavy (H) groups. The pre-exercise (pre-ex) and post-exercise (post-ex) data series were compared; Table S12: The right area of back musculature (ROI 4). Values (mean ± SD) of gray-level run-length matrix (GLRLM) features for three color components (R, red; G, green; B, blue) in light (L), moderate (M), and heavy (H) groups. The pre-exercise (pre-ex) and post-exercise (post-ex) data series were compared; Table S13: The withers area (ROI 1). Values (mean ± SD) of gray level co-occurrence matrix (GLCM) features for three color components (R, red; G, green; B, blue) in light (L), moderate (M), and heavy (H) groups. The pre-exercise (pre-ex) and post-exercise (post-ex) data series were compared; Table S14: The thoracic spine area (ROI 2). Values (mean ± SD) of gray level co-occurrence matrix (GLCM) features for three color components (R, red; G, green; B, blue) in light (L), moderate (M), and heavy (H) groups. The pre-exercise (pre-ex) and post-exercise (post-ex) data series were compared; Table S15: The left area of back musculature (ROI 3). Values (mean ± SD) of gray level co-occurrence matrix (GLCM) features for three color components (R, red; G, green; B, blue) in light (L), moderate (M), and heavy (H) groups. The pre-exercise (pre-ex) and post-exercise (post-ex) data series were compared; Table S16. The right area of back musculature (ROI 4). Values (mean ± SD) of gray level co-occurrence matrix (GLCM) features for three color components (R, red; G, green; B, blue) in light (L), moderate (M), and heavy (H) groups. The pre-exercise (pre-ex) and post-exercise (post-ex) data series were compared.

## References

[1] Han J., Lawler D., Kimm S. (2015). Childhood obesity. Lancet.

[2] Wang Y., McPherson K., March T., Gortmaker S., Brown M. (2017). Health and economic burden of the projected obesity trends in the USA and UK. Lancet.

[3] Forino S., Cameron L., Stones N., Freeman M. (2021). Potential Impacts of Body Image Perception in Female Equestrians. J. Equine Vet. Sci..

[4] Kozak M.W. (2017). Making trails: Horses and equestrian tourism in Poland. Equestrian Cultures in Global and Local Contexts.

[5] Matsuura A., Mano H., Irimajiri M., Hodate K. (2016). Maximum permissible load for Yonaguni ponies (Japanese landrace horses) trotting over a short, straight course. Anim. Welf..

[6] Garlinghouse S., Burill M. (1999). Relationship of body condition score to completion rate during 160 km endurance races. Equine Vet. J..

[7] Matsuura A., Sakuma S., Irimajiri M., Hodate K. (2013). Maximum permissible load weight of a Taishuh pony at a trot. J. Anim. Sci..

[8] Clayton H., Dyson S., Harris P., Bondi A. (2015). Horses, saddles and riders: Applying the science. Equine Vet. Educ..

[9] Hall C., Kay R., Randle H., Preshaw L., Pearson G., Waran N. Indicators on the outside: Behaviour and equine quality of life. Proceedings of the 15th International Conference of the International Society for Equitation Science.

[10] Randle H., Henshall C., Hall C., Pearson G., Preshaw L., Waran N. Indicators on the inside: Physiology and equine quality of life. Proceedings of the 15th International Conference of the International Society for Equitation Science.

[11] Hall C., Randle H., Pearson G., Preshaw L., Waran N. (2018). Assessing equine emotional state. Appl. Anim. Behav. Sci..

[12] Becker-Birck M., Schmidt A., Lasarzik J., Aurich J., Möstl E., Aurich C. (2013). Cortisol release and heart rate variability in sport horses participating in equestrian competitions. J. Vet. Behav..

[13] Waran N.K., Cuddeford D. (1995). Effects of loading and transport on the heart rate and behaviour of horses. Appl. Anim. Behav. Sci..

[14] Thayer J.F., Sternberg E. (2006). Beyond heart rate variability: Vagal regulation of allostatic systems. Ann. N. Y. Acad. Sci..

[15] Visser E.K., Van Reenen C.G., Van der Werf J.T.N., Schilder M.B.H., Knaap J.H., Barneveld A., Blokhuis H.J. (2002). Heart rate and heart rate variability during a novel object test and a handling test in young horses. Physiol. Behav..

[16] Waran N., Randle H. (2017). What we can measure, we can manage: The importance of using robust welfare indicators in Equitation Science. Appl. Anim. Behav. Sci..

[17] de Mira M.C., Lamy E., Santos R., Williams J., Pinto M.V., Martins P.S., Rodrigues P., Marlin D. (2021). Salivary cortisol and eye temperature changes during endurance competitions. BMC Vet. Res..

[18] Hall C., Burton K., Maycock E., Wragg E. (2011). A preliminary study into the use of infrared thermography as a means of assessing the horse’s response to different training methods. J. Vet. Behav..

[19] Redaelli V., Luzi F., Mazzola S., Bariffi G.D., Zappaterra M., Nanni Costa L., Padalino B. (2019). The use of infrared thermography (IRT) as stress indicator in horses trained for endurance: A pilot study. Animals.

[20] Travain T., Valsecchi P. (2021). Infrared Thermography in the Study of Animals’ Emotional Responses: A Critical Review. Animals.

[21] Soroko M., Howell K. (2018). Infrared thermography: Current applications in equine medicine. J. Equine Vet. Sci..

[22] Roberto J.V.B., De Souza B.B. (2020). Use of infrared thermography in veterinary medicine and animal production. J. Anim. Behav. Biometeorol..

[23] Witkowska-Piłaszewicz O., Maśko M., Domino M., Winnicka A. (2020). Infrared thermography correlates with lactate concentration in blood during race training in horses. Animals.

[24] Wilk I., Wnuk-Pawlak E., Janczarek I., Kaczmarek B., Dybczyńska M., Przetacznik M. (2020). Distribution of superficial body temperature in horses ridden by two riders with varied body weights. Animals.

[25] Masko M., Borowska M., Domino M., Jasinski T., Zdrojkowski L., Gajewski Z. (2021). A novel approach to thermographic images analysis of equine thoracolumbar region: The effect of effort and rider’s body weight on structural image complexity. BMC Vet. Res..

[26] Powell D., Bennett-Wimbush K., Peeples A., Duthie M. (2008). Evaluation of indicators of weight-carrying ability of light riding horses. J. Equine Vet. Sci..

[27] Christensen J.W., Bathellier S., Rhodin M., Palme R., Uldahl M. (2020). Increased rider weight did not induce changes in behavior and physiological parameters in horses. Animals.

[28] Christensen J.W., Beekmans M., van Dalum M., Van Dierendonck M. (2014). Effects of hyperflexion on acute stress responses in ridden dressage horses. Physiol. Behav..

[29] Zebisch A., May A., Reese S., Gehlen H. (2014). Effect of different head-neck positions on physical and psychological stress parameters in the ridden horse. J. Anim. Physiol. Anim. Nutr..

[30] Dyson S., Ellis A.D., Mackechnie-Guire R., Douglas J., Bondi A., Harris P. (2020). The influence of rider:horse bodyweight ratio and rider-horse-saddle fit on equine gait and behaviour: A pilot study. Equine Vet. Educ..

[31] Resmini R., Silva L., Araujo A.S., Medeiros P., Muchaluat-Saade D., Conci A. (2021). Combining Genetic Algorithms and SVM for Breast Cancer Diagnosis Using Infrared Thermography. Sensors.

[32] Depeursinge A., Al-Kadi O.S., Mitchell J.R. (2017). Biomedical Texture Analysis: Fundamentals, Tools and Challenges.

[33] Bębas E., Borowska M., Derlatka M., Oczeretko E., Hładuński M., Szumowski P., Mojsak M. (2021). Machine-learning-based classification of the histological subtype of non-small-cell lung cancer using MRI texture analysis. Biomed. Signal Process. Control.

[34] Sohail A.S.M., Bhattacharya P., Mudur S.P., Krishnamurthy S. Local relative GLRLM-based texture feature extraction for classifying ultrasound medical images. Proceedings of the 2011 24th Canadian Conference on Electrical and Computer Engineering (CCECE, IEEE).

[35] Girejko G., Borowska M., Szarmach J. (2018). Statistical analysis of radiographic textures illustrating healing process after the guided bone regeneration surgery. International Conference on Information Technologies in Biomedicine.

[36] Obuchowicz R., Nurzynska K., Obuchowicz B., Urbanik A., Piórkowski A. (2020). Caries detection enhancement using texture feature maps of intraoral radiographs. Oral Radiol..

[37] Pociask E., Nurzynska K., Obuchowicz R., Bałon P., Uryga D., Strzelecki M., Izworski A., Piórkowski A. (2021). Differential Diagnosis of Cysts and Granulomas Supported by Texture Analysis of Intraoral Radiographs. Sensors.

[38] Zhang H., Hung C.L., Min G., Guo J.P., Liu M., Hu X. (2019). GPU-accelerated GLRLM algorithm for feature extraction of MRI. Sci. Rep..

[39] Domino M., Borowska M., Kozłowska N., Zdrojkowski Ł., Jasiński T., Smyth G., Maśko M. (2022). Advances in Thermal Image Analysis for the Detection of Pregnancy in Horses Using Infrared Thermography. Sensors.

[40] Silva T.A.E., Silva L.F., Muchaluat-Saade D.C., Conci A. (2020). A computational method to assist the diagnosis of breast disease using dynamic thermography. Sensors.

[41] Martin B.B., Klide A.M. (1999). Physical examination of horses with back pain. Vet. Clin. N. Am. Equine Pract..

[42] Dyson S. (2011). Can lameness be reliably graded?. Equine Vet. J..

[43] Greve L., Dyson S. (2015). Saddle fit and management: An investigation of the association with equine thoracolumbar asymmetries, horse and rider health. Equine Vet. J..

[44] Williams J., Tabor G. (2017). Rider impacts on equitation. Appl. Anim. Behav. Sci..

[45] NIH (2018). Calculate Your Body Mass Index. https://www.nhlbi.nih.gov/health/educational/lose_wt/BMI/bmicalc.htm.

[46] McCafferty D.J. (2007). The value of infrared thermography for research on mammals: Previous applications and future directions. Mammal Rev..

[47] Szczypinski P.M., Klepaczko A., Kociołek M. (2017). Qmazda—Software tools for image analysis and pattern recognition. 2017 Signal Processing: Algorithms, Architectures, Arrangements, and Applications (SPA).

[48] Wen C.-Y., Chou C.-M. (2004). Color image models and its applications to document examination. Forensic Sci. J..

[49] Szczypinski P.M., Klepaczko A. (2017). Mazda—A framework for biomedical image texture analysis and data exploration. Biomedical Texture Analysis.

[50] Materka A., Strzelecki M. (1998). Texture Analysis Methods—A Review. COST B11 Report.

[51] Galloway M.M. (1975). Texture classification using gray level run length. Comput. Graph. Image Process..

[52] Tang X. (1998). Texture information in run-length matrices. IEEE Trans. Image Process..

[53] Haralick R.M. (1979). Statistical and structural approaches to texture. Proc. IEEE.

[54] Soroko M., Howell K., Dudek K. (2017). The effect of ambient temperature on infrared thermographic images of joints in the distal forelimbs of healthy racehorses. J. Therm. Biol..

[55] Hodgson D.R., Davis R.E., McConaghy F.F. (1994). Thermoregulation in the horse in response to exercise. Br. Vet. J..

[56] Ibraheem N.A., Hasan M.M., Khan R.Z., Mishra P.K. (2012). Understanding color models: A review. ARPN J. Sci. Technol..

[57] Plataniotis K.N., Venetsanopoulos A.N. (2013). Color Image Processing and Applications.

[58] Soroko M., Śpitalniak-Bajerska K., Zaborski D., Poźniak B., Dudek K., Janczarek I. (2019). Exercise-induced changes in skin temperature and blood parameters in horses. Arch. Anim. Breed..

[59] Maśko M., Zdrojkowski L., Domino M., Jasinski T., Gajewski Z. (2019). The Pattern of Superficial Body Temperatures in Leisure Horses Lunged with Commonly Used Lunging Aids. Animals.

[60] Borowska M. (2015). Entropy-based algorithms in the analysis of biomedical signals. Stud. Log. Gramm. Rhetor..

[61] Janczarek I., Wilk I. (2017). Leisure riding horses: Research topics versus the needs of stakeholders. Anim. Sci. J..

[62] Häyrynen T.A.H. (2019). Smart Phone Thermal Camera Accessory Device as a Mean to Asses Saddle Fit in Horses. Master’s Thesis.

[63] Kang H., Zsoldos R.R., Woldeyohannes S.M., Gaughan J.B., Sole Guitart A. (2020). The Use of Percutaneous Thermal Sensing Microchips for Body Temperature Measurements in Horses Prior to, during and after Treadmill Exercise. Animals.

[64] MacKechnie-Guire R., Fisher M., Mathie H., Kuczynska K., Fairfax V., Fisher D., Pfau T. (2021). A Systematic Approach to Comparing Thermal Activity of the Thoracic Region and Saddle Pressure Distribution beneath the Saddle in a Group of Non-Lame Sports Horses. Animals.

[65] Pereira N., Valenzuela D., Mangelsdorff G., Kufeke M., Roa R. (2018). Detection of perforators for free flap planning using smartphone thermal imaging: A concordance study with computed tomographic angiography in 120 perforators. Plast. Reconstr. Surg..

[66] Van Doremalen R.F.M., Van Netten J.J., Van Baal J.G., Vollenbroek-Hutten M.M.R., van der Heijden F. (2019). Validation of low-cost smartphone-based thermal camera for diabetic foot assessment. Diabetes Res. Clin. Pract..

[67] Jaiswal A., Amjad Z., Jha S., Sahni N., Chirayil S.B., Nair R.C. (2021). Accurate Device Temperature Forecasting using Recurrent Neural Network for Smartphone Thermal Management. Proceedings of the 2021 International Joint Conference on Neural Networks (IJCNN).

[68] Soroko M., Cwynar P., Howell K., Yarnell K., Dudek K., Zaborski D. (2018). Assessment of saddle fit in racehorses using infrared thermography. J. Equine Vet. Sci..

