# Supplementary material for: The Effect of Rider:Horse Bodyweight Ratio on the Superficial Body Temperature of Horse’s Thoracolumbar Region Evaluated by Advanced Thermal Image Processing
Source: Animals (Basel). 2022 Jan 13;12(2):195. doi: 10.3390/ani12020195 (PMC8772910; doi:10.3390/ani12020195)
Supplement: Supplementary file 1 [file animals-12-00195-s001.zip › animals-1527327-supplementary.pdf]

Supplementary Data for

The Effect of Rider:Horse Bodyweight Ratio on the Superficial Body Temperature of Horse's Thoracolumbar Region Evaluated by Advanced Thermal Image Processing

Małgorzata Domino [1], Marta Borowska [2], Anna Trojakowska [3], Natalia Kozłowska [1], Łukasz Zdrojkowski [1,*], Tomasz Jasiński [1] Graham Smyth[4], and Małgorzata Maśko [5,*]

1 Department of Large Animal Diseases and Clinic, Institute of Veterinary Medicine, Warsaw University of Life Sciences (WULS–SGGW), Warsaw, 02–787, Poland; malgorzata_domino@sggw.edu.pl (M.D.); natalia_kozlowska@sggw.edu.pl (N.K.); tomasz_jasinski@sggw.edu.pl (T.J.)

2 Institute of Biomedical Engineering, Faculty of Mechanical Engineering, Białystok University of Technology, Bialystok, 15-351, Poland; m.borowska@pb.edu.pl (M.B.)

3 The Scientific Society of Veterinary Medicine Students, Warsaw University of Life Sciences, Warsaw, 02-787, Poland; an.trojakowska@gmail.com (A.T.)

4 Menzies Health Institute Queensland and Griffith University School of Medicine, Southport, QLD, 4222, Australia; grahamcsmyth@gmail.com (G.S.)

5 Department of Animal Breeding, Institute of Animal Science, Warsaw University of Life Sciences (WULS–SGGW), Warsaw, 02–787, Poland

* Correspondence: lukasz_zdrojkowski@sggw.edu.pl (Ł.Z.); malgorzata_masko@sggw.edu.pl (M.M.)

Table S1. The withers area (ROI 1)**.** Values (mean ± SD) of conventional thermal features in light (L), moderate (M), and heavy (H) groups. The pre-exercise (pre-ex) and post exercise (post-ex) data series were compared. When features differed significantly (p<0.05) for all three groups (L, M, H), the values were marked in bold.

Groups		Taver	Tmax	Tmin
	pre-ex	**27.5±2.8**	**31.1±2.6**	**24.6±3.2**
L	post-ex	**35.7±2.2**	**38.1±2.0**	**32.2±3.0**
	p	**< 0.0001**	**< 0.0001**	**< 0.0001**
	pre-ex	**26.9±3.3**	**31.2±3.5**	**24.0±3.7**
M	post-ex	**36.6±2.4**	**39.0±2.2**	**33.1±2.4**
	p	**< 0.0001**	**< 0.0001**	**< 0.0001**
	pre-ex	**26.2±2.8**	**30.3±3.0**	**23.4±3.1**
H	post-ex	**36.3±2.1**	**39.1±2.0**	**32.7±3.0**
	p	**< 0.0001**	**< 0.0001**	**< 0.0001**

ROI - regions of interest. Taver - the average temperature, Tmax - the maximal temperature, Tmin - the minimal temperature.

Table S2. The thoracic spine area (ROI 2)**.** Values (mean ± SD) of conventional thermal features in light (L), moderate (M), and heavy (H) groups. The pre-exercise (pre-ex) and post exercise (post-ex) data series were compared. When features differed significantly (p<0.05) for all three groups (L, M, H), the values were marked in bold.

Groups		Taver	Tmax	Tmin
	pre-ex	**29.7±2.5**	**33.0±2.3**	**26.5±3.0**
L	post-ex	**35.2±2.0**	**37.9±2.0**	**32.1±3.0**
	p	**< 0.0001**	**< 0.0001**	**< 0.0001**
	pre-ex	**29.3±3.0**	**32.5±3.1**	**25.7±3.4**
M	post-ex	**35.9±2.7**	**38.5±2.8**	**33.3±2.7**
	p	**< 0.0001**	**< 0.0001**	**< 0.0001**
	pre-ex	**28.6±2.7**	**32.2±3.2**	**25.0±3.0**
H	post-ex	**35.8±2.2**	**38.9±2.3**	**32.8±2.7**
	p	**< 0.0001**	**< 0.0001**	**< 0.0001**

ROI - regions of interest. Taver - the average temperature, Tmax - the maximal temperature, Tmin - the minimal temperature.

Table S3. The left area of back musculature (ROI 3). Values (mean ± SD) of conventional thermal features in light (L), moderate (M), and heavy (H) groups. The pre-exercise (pre-ex) and post exercise (post-ex) data series were compared. When features differed significantly (p<0.05) for all three groups (L, M, H), the values were marked in bold.

Groups		Taver	Tmax	Tmin
L	pre-ex	**26.6±3.0**	**31.9±2.7**	**22.8±3.3**
	post-ex	**35.2±2.5**	**38.3±1.9**	**30.5±3.2**
	p	**< 0.0001**	**< 0.0001**	**< 0.0001**
M	pre-ex	**26.2±3.7**	**31.8±3.3**	**22.4±4.4**
	post-ex	**36.1±2.6**	**39.2±2.3**	**31.9±2.8**
	p	**< 0.0001**	**< 0.0001**	**< 0.0001**
H	pre-ex	**25.8±3.0**	**31.7±3.2**	**22.0±3.3**
	post-ex	**35.9±2.3**	**39.3±2.1**	**31.5±2.6**
	p	**< 0.0001**	**< 0.0001**	**< 0.0001**

ROI - regions of interest. Taver - the average temperature, Tmax - the maximal temperature, Tmin - the minimal temperature.

Table S4. The right area of back musculature (ROI 4). Values (mean ± SD) of conventional thermal features in light (L), moderate (M), and heavy (H) groups. The pre-exercise (pre-ex) and post exercise (post-ex) data series were compared. When features differed significantly (p<0.05) for all three groups (L, M, H), the values were marked in bold.

Groups		Taver	Tmax	Tmin
L	pre-ex	**26.9±2.9**	**31.9±2.8**	**23.5±3.3**
	post-ex	**35.2±2.5**	**38.3±1.9**	**31.0±3.1**
	p	**< 0.0001**	**< 0.0001**	**< 0.0001**
M	pre-ex	**26.4±3.6**	**32.0±3.3**	**22.8±4.1**
	post-ex	**36.3±2.7**	**38.9±2.6**	**32.1±2.9**
	p	**< 0.0001**	**< 0.0001**	**< 0.0001**
H	pre-ex	**26.0±3.0**	**32.1±3.4**	**22.2±3.2**
	post-ex	**36.1±2.2**	**39.7±1.8**	**31.0±3.4**
	p	**< 0.0001**	**< 0.0001**	**< 0.0001**

ROI - regions of interest. Taver - the average temperature, Tmax - the maximal temperature, Tmin - the minimal temperature.

Table S5. The withers area (ROI 1)**.** Values (mean ± SD) of Histogram Statistics (HS) features for three color components (R, red; G, green; B, blue) in light (L), moderate (M), and heavy (H) groups. The pre-exercise (pre-ex) and post exercise (post-ex) data series were compared. When features differed significantly (p<0.05) for all three groups (L, M, H), the values were marked in bold.

	Groups		Mean	Variance	Skewness	Kurtosis	Perc01	Perc10	Perc50	Perc90	Perc99	Maxm01	Domn01	Maxm10	Domn10
		pre-ex	**25.1±4.3**	**48.8±38.3**	**1.9±1.5**	**7.4±14.8**	**7.1±8.3**	**15.9±9.2**	**27.0±4.6**	30.8±0.5	31.0±0.0	**0.3±0.2**	29.6±6.3	**0.9±0.2**	15.0±0.2
	L	post-ex	**29.9±0.8**	**8.6±10.1**	**5.3±2.3**	**42.4±36.8**	**18.1±8.7**	**28.2±2.5**	**30.8±0.4**	31.0±0.0	31.0±0.0	**0.6±0.1**	31.0±0.0	**1.0±0.0**	15.0±0.0
		p	**< 0.0001**	**< 0.0001**	**< 0.0001**	**< 0.0001**	**< 0.0001**	**< 0.0001**	**< 0.0001**	0.250	>0.999	**< 0.0001**	0.250	**< 0.0001**	>0.999
		pre-ex	**24.5±4.1**	**57.0±41.5**	**1.7±1.3**	**4.6±7.8**	**4.5±6.6**	**14.5±9.2**	**26.8±3.9**	30.8±0.5	31.0±0.0	**0.3±0.2**	28.0±8.7	**0.8±0.1**	15.0±0.0
**R**	M	post-ex	**29.2±1.5**	**15.7±17.8**	**4.5±2.3**	**32.2±31.9**	**14.2±9.1**	**25.9±5.4**	**30.6±0.6**	31.0±0.0	31.0±0.0	**0.6±0.1**	31.0±0.0	**1.0±0.0**	15.0±0.0
		p	**< 0.0001**	**< 0.0001**	**< 0.0001**	**< 0.0001**	**< 0.0001**	**< 0.0001**	**< 0.0001**	0.250	>0.999	**< 0.0001**	0.100	**< 0.0001**	>0.999
		pre-ex	**23.5±3.7**	**63.0±39.1**	**1.2±0.7**	**1.4±3.6**	**4.3±6.5**	**12.1±8.1**	**25.6±3.7**	30.8±0.4	31.0±0.0	**0.2±0.1**	29.4±6.3	**0.8±0.1**	15.0±0.0
	H	post-ex	**27.6±3.0**	**33.5±37.8**	**2.8±1.3**	**12.1±11.6**	**10.6±8.7**	**20.8±9.1**	**29.6±1.7**	31.0±0.0	31.0±0.0	**0.4±0.1**	31.0±0.2	**0.9±0.1**	15.0±0.0
		p	**< 0.0001**	**0.006**	**< 0.0001**	**0.0001**	**< 0.0001**	**< 0.0001**	**< 0.0001**	0.125	>0.999	**< 0.0001**	0.094	**0.0003**	>0.999
		pre-ex	**21.6±2.0**	**25.2±16.9**	**1.5±0.6**	**2.4±2.8**	**7.3±4.1**	**14.5±4.9**	**23.2±1.8**	25.9±0.3	26.0±0.0	**0.2±0.1**	25.8±0.6	**0.9±0.1**	10.0±0.0
	L	post-ex	**13.6±3.0**	**61.1±14.1**	**0.0±0.6**	**-1.1±0.6**	**3.0±0.0**	**3.4±1.1**	**13.8±5.1**	23.8±2.5	25.8±0.8	**0.2±0.1**	6.8±8.8	**0.7±0.1**	9.6±4.0
		p	**< 0.0001**	**< 0.0001**	**< 0.0001**	**< 0.0001**	**< 0.0001**	**< 0.0001**	**< 0.0001**	0.0001	0.250	**0.034**	< 0.0001	**< 0.0001**	0.500
		pre-ex	**21.8±1.9**	**23.1±15.5**	**1.4±0.6**	**2.3±3.0**	**8.5±4.9**	**15.0±4.8**	**23.5±1.4**	26.0±0.0	26.0±0.0	**0.3±0.1**	25.9±0.4	**0.9±0.1**	10.0±0.0
**G**	M	post-ex	**14.6±3.6**	**62.6±12.5**	**0.1±0.7**	**-1.0±0.7**	**3.0±0.0**	**3.9±2.1**	**15.1±6.2**	24.4±2.0	26.0±0.0	**0.2±0.1**	10.7±11.1	**0.7±0.1**	9.0±4.1
		p	**< 0.0001**	**< 0.0001**	**< 0.0001**	**< 0.0001**	**< 0.0001**	**< 0.0001**	**< 0.0001**	< 0.0001	>0.999	**0.001**	0.130	**< 0.0001**	0.322
		pre-ex	**22.2±1.2**	**21.3±12.3**	**1.6±0.6**	**2.8±3.3**	**7.9±4.5**	**15.9±3.8**	**24.0±1.1**	26.0±0.0	26.0±0.0	**0.3±0.1**	26.0±0.5	**1.0±0.0**	10.0±0.0
	H	post-ex	**17.6±2.5**	**61.5±17.2**	**0.7±0.5**	**-0.6±1.0**	**3.0±0.4**	**5.3±3.0**	**20.0±3.5**	25.8±0.7	26.0±0.0	**0.2±0.0**	18.3±11.1	**0.8±0.1**	9.3±2.3
		p	**< 0.0001**	**< 0.0001**	**< 0.0001**	**< 0.0001**	**< 0.0001**	**< 0.0001**	**< 0.0001**	0.250	>0.999	**< 0.0001**	0.078	**< 0.0001**	0.500
		pre-ex	**2.4±1.6**	16.5±20.2	**-2.3±1.2**	**9.9±14.3**	0.0±0.0	0.0±0.0	**1.0±0.9**	**6.9±5.6**	**15.1±9.1**	**0.4±0.1**	0.1±0.3	**0.6±0.1**	0.0±0.0
	L	post-ex	**4.0±0.9**	8.2±2.8	**-0.6±0.8**	**1.0±3.9**	0.0±0.2	0.7±0.8	**3.7±1.4**	**7.9±1.0**	**11.0±2.9**	**0.2±0.0**	2.0±2.4	**0.9±0.1**	0.0±0.0
		p	**< 0.0001**	0.0004	**< 0.0001**	**< 0.0001**	>0.999	0.0004	**< 0.0001**	**< 0.0001**	**< 0.0001**	**< 0.0001**	0.0002	**< 0.0001**	-
		pre-ex	**2.5±1.9**	19.6±24.8	**-2.7±1.3**	**11.0±10.8**	0.0±0.0	0.0±0.0	**0.9±0.8**	**7.5±6.5**	**16.5±7.7**	**0.4±0.1**	0.1±0.3	**0.5±0.1**	0.0±0.0
**B**	M	post-ex	**3.9±1.2**	9.9±4.0	**-0.9±1.0**	**2.0±5.2**	0.0±0.0	0.5±0.7	**3.3±1.6**	**8.1±1.4**	**12.3±4.8**	**0.2±0.1**	2.5±3.1	**0.8±0.1**	0.0±0.0
		p	**< 0.0001**	0.143	**< 0.0001**	**< 0.0001**	-	0.0004	**< 0.0001**	**0.023**	**< 0.0001**	**< 0.0001**	< 0.0001	**< 0.0001**	-
		pre-ex	**2.5±1.7**	22.2±22.4	**-2.6±1.1**	**10.2±10.1**	0.0±0.0	0.0±0.0	**0.8±0.8**	**7.8±5.8**	**18.3±8.7**	**0.5±0.1**	0.0±0.0	**0.5±0.1**	0.0±0.0
	H	post-ex	**3.3±0.9**	14.8±12.4	**-1.4±0.9**	**3.1±5.3**	0.0±0.0	0.0±0.2	**2.0±1.0**	**8.3±2.9**	**14.1±6.5**	**0.3±0.1**	0.4±1.5	**0.7±0.1**	0.0±0.0
		p	**< 0.0001**	0.360	**< 0.0001**	**0.002**	-	>0.999	**< 0.0001**	**< 0.0001**	**< 0.0001**	**< 0.0001**	0.250	**< 0.0001**	-

R - Red component; G - Green component; B - Blue component. Skewness - skewness coefficient; Perc01, Perc10, Perc50, Perc90, Perc99 - percentiles; Domn01, Domn10 - dominants; Maxm01, Maxm10 - maximum of moments; p - the level of marginal significance; SD - standard deviation.

Table S6. The thoracic spine area (ROI 2). Values (mean ± SD) of Histogram Statistics (HS) features for three color components (R, red; G, green; B, blue) in light (L), moderate (M), and heavy (H) groups. The pre-exercise (pre-ex) and post exercise (post-ex) data series were compared. When features differed significantly (p<0.05) for all three groups (L, M, H), the values were marked in bold.

Groups			Mean	Variance	Skewness	Kurtosis	Perc01	Perc10	Perc50	Perc90	Perc99	Maxm01	Domn01	Maxm10	Domn10
		pre-ex	29.7±1.7	12.7±21.1	3.7±1.4	22.2±23.0	19.3±10.7	26.6±5.7	30.8±0.7	31.0±0.0	31.0±0.0	0.7±0.2	31.0±0.0	1.0±0.0	15.0±0.0
	L	post-ex	30.3±0.8	3.3±4.9	4.1±2.0	28.7±29.5	24.4±5.7	28.8±2.2	30.9±0.3	31.0±0.0	31.0±0.0	0.7±0.2	31.0±0.0	1.0±0.0	15.0±0.0
		p	0.330	0.059	0.833	0.623	0.078	0.063	0.750	>0.999	>0.999	0.317	>0.999	0.095	>0.999
		pre-ex	29.3±1.8	19.3±28.8	3.6±1.6	20.8±27.3	16.1±10.6	24.9±7.2	30.9±0.3	31.0±0.0	31.0±0.0	0.7±0.1	31.0±0.0	1.0±0.1	15.0±0.0
**R**	M	post-ex	29.6±1.4	7.0±9.1	3.4±1.4	17.4±13.8	20.9±7.0	27.0±3.8	30.4±1.1	31.0±0.0	31.0±0.0	0.6±0.2	31.0±0.0	1.0±0.0	15.0±0.0
		p	0.491	0.054	0.989	0.833	0.056	0.500	0.063	>0.999	>0.999	0.241	>0.999	0.063	>0.999
		pre-ex	29.2±1.7	18.7±26.1	4.0±2.1	25.8±34.1	15.1±10.6	25.1±6.8	30.8±0.5	31.0±0.0	31.0±0.0	0.7±0.2	31.0±0.0	1.0±0.0	15.0±0.0
	H	post-ex	29.1±1.9	12.5±17.0	3.7±3.1	30.4±51.7	18.8±9.9	25.6±5.5	30.2±1.1	31.0±0.0	31.0±0.0	0.5±0.3	30.9±0.3	1.0±0.0	15.0±0.0
		p	0.944	0.264	0.406	0.623	0.102	0.979	0.077	>0.999	>0.999	0.197	0.500	>0.999	>0.999
		pre-ex	**13.9±3.0**	**57.1±13.6**	**0.0±0.5**	**-1.1±0.6**	3.1±0.4	**3.6±1.4**	**14.1±4.8**	23.6±2.6	25.7±1.1	0.2±0.1	9.4±10.3	**0.7±0.1**	5.4±3.9
	L	post-ex	**16.9±3.2**	**39.0±10.0**	**0.7±0.7**	**0.0±1.8**	3.2±0.8	**7.6±4.1**	**18.0±4.0**	24.0±1.8	25.8±0.5	0.1±0.1	18.4±8.6	**0.8±0.1**	8.2±2.8
		p	**0.0004**	**< 0.0001**	**0.001**	**< 0.0001**	0.750	**< 0.0001**	**0.022**	0.504	>0.999	0.084	0.0007	**0.005**	0.002
		pre-ex	**14.7±3.2**	**57.0±15.8**	**0.1±0.6**	**-0.9±1.0**	3.2±0.7	**4.7±3.5**	**15.1±4.7**	24.5±1.8	25.8±0.5	0.2±0.1	9.9±10.6	**0.7±0.1**	6.3±3.9
**G**	M	post-ex	**18.0±4.0**	**38.6±14.8**	**0.9±0.8**	**0.7±2.0**	3.7±2.1	**8.7±4.9**	**19.5±4.9**	24.5±2.9	25.6±1.5	0.2±0.1	19.5±9.0	**0.8±0.1**	8.8±2.7
		p	**0.004**	**0.0004**	**0.001**	**0.0003**	0.336	**0.003**	**0.002**	>0.999	0.625	>0.999	0.006	**0.0006**	0.021
		pre-ex	**15.7±3.7**	**49.9±15.8**	**0.3±0.7**	**-0.6±1.1**	3.4±1.1	**6.0±4.0**	**16.2±5.1**	24.6±2.1	26.0±0.2	0.2±0.1	15.5±11.3	**0.8±0.1**	6.5±4.0
	H	post-ex	**18.6±4.1**	**35.4±11.7**	**1.0±1.1**	**1.3±2.4**	3.4±1.1	**9.7±4.4**	**20.0±5.1**	24.3±3.4	25.8±0.5	0.2±0.1	23.3±5.3	**0.9±0.1**	8.7±2.5
		p	**0.003**	**0.004**	**0.002**	**0.0008**	0.953	**0.004**	**0.002**	0.822	0.125	0.095	0.130	**0.002**	0.064
		pre-ex	**3.8±0.8**	**6.9±4.6**	-0.5±0.8	0.6±3.1	0.1±0.4	0.8±0.9	**3.6±1.1**	**7.0±0.8**	10.0±4.1	**0.2±0.1**	2.9±2.7	0.9±0.1	0.0±0.0
	L	post-ex	**2.8±1.0**	**4.0±1.5**	-1.1±0.9	2.9±6.1	0.0±0.2	0.6±0.6	**2.5±1.1**	**5.4±1.4**	8.2±1.4	**0.3±0.1**	1.6±1.2	0.9±0.1	0.0±0.0
		p	**0.0004**	**< 0.0001**	0.002	0.011	0.750	0.449	**0.002**	**< 0.0001**	0.078	**0.005**	0.014	0.406	-
		pre-ex	**3.7±1.0**	**9.3±9.9**	-0.7±1.0	1.6±4.4	0.0±0.0	0.5±0.7	**3.4±1.2**	**7.5±2.9**	11.1±5.7	**0.2±0.1**	2.0±2.3	0.8±0.1	0.0±0.0
**B**	M	post-ex	**2.5±1.2**	**4.2±1.8**	-1.2±0.9	3.2±4.8	0.1±0.4	0.5±0.9	**1.9±1.5**	**5.2±1.5**	8.3±2.1	**0.3±0.1**	1.5±1.9	0.8±0.2	0.0±0.0
		p	**< 0.0001**	**< 0.0001**	0.027	0.056	0.500	>0.999	**0.0008**	**0.0002**	0.002	**0.0008**	0.491	0.303	-
		pre-ex	**3.3±1.1**	**8.2±8.8**	-1.1±1.6	5.0±9.3	0.0±0.0	0.5±0.7	**2.9±1.3**	**6.8±2.7**	11.1±5.7	**0.2±0.1**	1.8±2.4	0.8±0.1	0.0±0.0
	H	post-ex	**2.4±1.2**	**5.1±3.4**	-1.5±1.3	5.2±10.0	0.0±0.2	0.5±1.0	**1.9±1.6**	**5.3±1.4**	9.6±4.1	**0.4±0.1**	1.2±1.8	0.7±0.2	0.0±0.0
		p	**< 0.0001**	**< 0.0001**	0.208	0.473	>0.999	0.781	**0.003**	**0.024**	0.094	**0.0002**	0.116	0.056	-

R - Red component; G - Green component; B - Blue component. Skewness - skewness coefficient; Perc01, Perc10, Perc50, Perc90, Perc99 - percentiles; Domn01, Domn10 - dominants; Maxm01, Maxm10 - maximum of moments; p - the level of marginal significance; SD - standard deviation.

Table S7. The left area of back musculature (ROI 3). Values (mean ± SD) of Histogram Statistics (HS) features for three color components (R, red; G, green; B, blue) in light (L), moderate (M), and heavy (H) groups. The pre-exercise (pre-ex) and post exercise (post-ex) data series were compared. When features differed significantly (p<0.05) for all three groups (L, M, H), the values were marked in bold.

Groups			Mean	Variance	Skewness	Kurtosis	Perc01	Perc10	Perc50	Perc90	Perc99	Maxm01	Domn01	Maxm10	Domn10
		pre-ex	**19.6±5.9**	**102.1±51.6**	**0.7±1.0**	**0.2±2.9**	**2.5±5.9**	**6.5±9.4**	**21.1±8.1**	30.7±0.9	31.0±0.0	**0.3±0.1**	**19.4±15.3**	**0.7±0.2**	14.4±3.1
	L	post-ex	**29.7±1.2**	**11.2±16.1**	**4.0±2.0**	**33.8±32.1**	**19.3±10.5**	**26.9±4.5**	**30.8±0.4**	31.0±0.0	31.0±0.0	**0.6±0.1**	**31.0±0.2**	**1.0±0.0**	15.0±0.0
		p	**< 0.0001**	**< 0.0001**	**< 0.0001**	**< 0.0001**	**< 0.0001**	**< 0.0001**	**< 0.0001**	0.125	>0.999	**< 0.0001**	**0.020**	**< 0.0001**	>0.999
		pre-ex	**20.0±4.7**	**96.8±42.3**	**0.7±0.8**	**0.0±2.3**	**1.2±3.1**	**6.3±7.1**	**21.6±6.5**	30.8±0.4	31.0±0.0	**0.2±0.1**	**22.0±14.3**	**0.7±0.2**	13.8±4.2
**R**	M	post-ex	**29.5±1.0**	**12.9±16.2**	**3.9±1.5**	**23.1±19.8**	**16.5±7.4**	**26.1±4.3**	**30.8±0.4**	31.0±0.0	31.0±0.0	**0.6±0.1**	**31.0±0.0**	**1.0±0.0**	15.0±0.0
		p	**< 0.0001**	**< 0.0001**	**< 0.0001**	**< 0.0001**	**< 0.0001**	**< 0.0001**	**< 0.0001**	0.125	>0.999	**< 0.0001**	**< 0.0001**	**< 0.0001**	0.500
		pre-ex	**19.5±5.5**	**95.9±41.4**	**0.6±0.8**	**-0.2±1.6**	**1.6±3.1**	**6.0±6.8**	**21.3±7.8**	30.4±1.9	31.0±0.2	**0.2±0.1**	**19.3±15.3**	**0.7±0.2**	13.8±4.2
	H	post-ex	**28.6±2.0**	**25.1±31.7**	**3.6±2.1**	**24.8±32.7**	**13.6±9.9**	**23.0±7.8**	**30.5±0.5**	31.0±0.0	31.0±0.0	**0.5±0.1**	**31.0±0.2**	**1.0±0.1**	15.0±0.0
		p	**< 0.0001**	**< 0.0001**	**< 0.0001**	**0.0004**	**< 0.0001**	**< 0.0001**	**< 0.0001**	0.011	0.303	**< 0.0001**	**0.0008**	**< 0.0001**	0.107
		pre-ex	**20.9±1.5**	**26.2±11.1**	**1.1±0.6**	**1.2±2.2**	**6.7±3.0**	**13.7±3.4**	**22.3±1.8**	**26.0±0.2**	26.0±0.0	0.2±0.1	**25.6±2.0**	**0.9±0.0**	**10.0±0.0**
	L	post-ex	**12.6±3.5**	**62.4±13.8**	**-0.3±0.7**	**-0.9±1.0**	**3.0±0.0**	**3.2±0.7**	**11.7±5.9**	**23.7±2.9**	25.6±0.8	0.3±0.1	**7.8±9.5**	**0.7±0.1**	**4.7±3.9**
		p	**< 0.0001**	**< 0.0001**	**< 0.0001**	**< 0.0001**	**< 0.0001**	**< 0.0001**	**< 0.0001**	**< 0.0001**	0.094	0.125	**< 0.0001**	**< 0.0001**	**< 0.0001**
		pre-ex	**21.6±1.4**	**22.5±9.7**	**1.5±0.7**	**2.9±3.2**	**6.3±3.3**	**15.2±3.2**	**23.0±1.7**	**26.0±0.0**	26.0±0.0	0.2±0.1	**26.0±0.0**	**1.0±0.0**	**10.0±0.0**
**G**	M	post-ex	**14.3±2.2**	**73.4±8.8**	**0.1±0.4**	**-1.4±0.3**	**3.0±0.0**	**3.1±0.4**	**14.5±4.3**	**25.5±0.8**	26.0±0.0	0.2±0.1	**7.8±9.5**	**0.7±0.1**	**5.7±4.1**
		p	**< 0.0001**	**< 0.0001**	**< 0.0001**	**< 0.0001**	**< 0.0001**	**< 0.0001**	**< 0.0001**	**0.0002**	>0.999	0.491	**< 0.0001**	**< 0.0001**	**< 0.0001**
		pre-ex	**21.4±1.5**	**23.9±9.2**	**1.3±0.7**	**2.0±2.7**	**6.8±3.3**	**14.5±3.2**	**22.9±2.0**	**26.0±0.2**	26.0±0.0	0.2±0.1	**25.4±3.1**	**1.0±0.0**	**10.0±0.0**
	H	post-ex	**14.9±3.8**	**63.7±15.7**	**0.1±0.8**	**-0.8±1.1**	**3.0±0.2**	**3.9±1.5**	**16.0±6.2**	**24.6±3.0**	25.8±0.8	0.2±0.1	**13.5±11.7**	**0.7±0.1**	**7.0±4.0**
		p	**< 0.0001**	**< 0.0001**	**< 0.0001**	**< 0.0001**	**< 0.0001**	**< 0.0001**	**< 0.0001**	**0.020**	0.322	0.330	**< 0.0001**	**< 0.0001**	**0.0004**
		pre-ex	5.7±3.7	**58.9±43.6**	**-1.4±0.9**	2.3±4.7	0.0±0.0	0.0±0.0	2.8±3.8	16.7±9.5	**21.7±7.9**	**0.4±0.1**	**0.0±0.0**	**0.5±0.1**	1.4±3.7
	L	post-ex	4.5±1.3	**9.1±2.6**	**-0.4±0.7**	0.4±2.1	0.1±0.3	0.7±1.0	4.5±1.8	8.5±1.2	**11.5±2.6**	**0.2±0.1**	**3.4±3.5**	**0.9±0.1**	0.0±0.0
		p	0.527	**0.004**	**< 0.0001**	0.208	0.500	0.0008	< 0.0001	0.027	**< 0.0001**	**< 0.0001**	**< 0.0001**	**< 0.0001**	0.250
		pre-ex	4.6±3.2	**45.8±37.3**	**-2.2±1.4**	6.8±10.4	0.0±0.0	0.0±0.0	1.8±1.9	14.0±8.7	**22.1±5.8**	**0.4±0.1**	**0.0±0.0**	**0.5±0.1**	0.9±3.1
**B**	M	post-ex	3.9±0.6	**10.3±3.0**	**-0.6±0.8**	0.7±3.8	0.0±0.0	0.1±0.3	3.4±1.0	8.0±0.7	**11.7±3.9**	**0.2±0.1**	**1.7±2.9**	**0.8±0.1**	0.0±0.0
		p	0.406	**0.0004**	**< 0.0001**	0.002	-	0.250	< 0.0001	< 0.0001	**< 0.0001**	**< 0.0001**	**0.0004**	**< 0.0001**	0.500
		pre-ex	5.2±3.9	**52.2±43.0**	**-1.7±1.0**	3.4±4.8	0.0±0.0	0.0±0.0	2.3±4.1	14.9±9.4	**21.4±7.6**	**0.4±0.1**	**0.0±0.0**	**0.5±0.1**	0.9±3.1
	H	post-ex	3.9±1.3	**12.7±9.9**	**-0.9±0.9**	1.4±3.4	0.0±0.2	0.4±1.0	3.3±1.7	8.3±2.1	**13.4±6.1**	**0.2±0.1**	**1.5±2.8**	**0.8±0.1**	0.0±0.0
		p	0.100	**< 0.0001**	**0.022**	>0.999	0.406	0.110	0.277	0.070	**0.0004**	**< 0.0001**	**0.002**	**< 0.0001**	0.500

R - Red component; G - Green component; B - Blue component. Skewness - skewness coefficient; Perc01, Perc10, Perc50, Perc90, Perc99 - percentiles; Domn01, Domn10 - dominants; Maxm01, Maxm10 - maximum of moments; p - the level of marginal significance; SD - standard deviation.

Table S8. The right area of back musculature (ROI 4)**.** Values (mean ± SD) of Histogram Statistics (HS) features for three color components (R, red; G, green; B, blue) in light (L), moderate (M), and heavy (H) groups. The pre-exercise (pre-ex) and post exercise (post-ex) data series were compared. When features differed significantly (p<0.05) for all three groups (L, M, H), the values were marked in bold.

**Groups**			**Mean**	**Variance**	**Skewness**	**Kurtosis**	**Perc01**	**Perc10**	**Perc50**	**Perc90**	**Perc99**	**Maxm01**	**Domn01**	**Maxm10**	**Domn10**
		pre-ex	**19.4±5.6**	**100.3±47.6**	**0.6±0.9**	**0.0±2.5**	**2.6±5.6**	**5.9±8.7**	**20.6±7.9**	**30.5±0.8**	31.0±0.0	**0.2±0.1**	**20.5±14.8**	**0.7±0.2**	14.3±3.1
	L	post-ex	**29.6±1.3**	**11.3±16.5**	**4.5±1.9**	**32.7±35.2**	**17.2±8.2**	**27.0±4.6**	**30.8±0.5**	**31.0±0.0**	31.0±0.0	**0.6±0.1**	**31.0±0.0**	**1.0±0.0**	15.0±0.0
		p	**< 0.0001**	**< 0.0001**	**< 0.0001**	**< 0.0001**	**< 0.0001**	**< 0.0001**	**< 0.0001**	**0.016**	>0.999	**< 0.0001**	**0.002**	**< 0.0001**	0.500
		pre-ex	**18.6±5.2**	**100.6±39.9**	**0.5±0.9**	**-0.3±2.8**	**1.8±5.1**	**4.8±7.6**	**19.7±7.6**	**30.3±1.0**	31.0±0.0	**0.2±0.1**	**20.4±14.7**	**0.6±0.2**	13.7±4.2
**R**	M	post-ex	**29.4±1.0**	**15.6±14.0**	**4.6±1.9**	**32.1±30.7**	**13.2±8.5**	**26.0±4.0**	**30.7±0.5**	**31.0±0.0**	31.0±0.0	**0.6±0.1**	**31.0±0.0**	**1.0±0.0**	15.0±0.0
		p	**< 0.0001**	**< 0.0001**	**< 0.0001**	**< 0.0001**	**< 0.0001**	**< 0.0001**	**< 0.0001**	**0.002**	>0.999	**< 0.0001**	**0.001**	**< 0.0001**	0.250
		pre-ex	**18.3±4.6**	**102.3±39.1**	**0.4±0.7**	**-0.5±2.0**	**1.2±3.5**	**4.5±6.5**	**19.1±6.3**	**30.4±1.2**	31.0±0.2	**0.2±0.1**	**18.0±15.5**	**0.6±0.2**	14.3±3.1
	H	post-ex	**29.1±1.6**	**20.0±25.4**	**4.9±3.0**	**45.1±62.9**	**14.1±9.9**	**25.0±6.7**	**30.5±0.5**	**31.0±0.0**	31.0±0.0	**0.5±0.1**	**31.0±0.2**	**1.0±0.0**	15.0±0.0
		p	**< 0.0001**	**< 0.0001**	**< 0.0001**	**< 0.0001**	**< 0.0001**	**< 0.0001**	**< 0.0001**	**0.008**	>0.999	**< 0.0001**	**< 0.0001**	**< 0.0001**	0.500
		pre-ex	**21.2±1.5**	**24.9±11.5**	**1.2±0.5**	**1.5±1.8**	**6.6±4.4**	**14.1±3.7**	**22.5±1.7**	**26.0±0.0**	26.0±0.0	0.2±0.1	**26.0±0.0**	**1.0±0.0**	**10.0±0.0**
	L	post-ex	**13.4±3.3**	**64.5±12.4**	**-0.1±0.7**	**-1.0±0.7**	**3.0±0.0**	**3.5±1.1**	**12.5±5.4**	**24.4±2.2**	25.9±0.6	0.2±0.1	**8.8±10.2**	**0.7±0.1**	**4.3±3.7**
		p	**< 0.0001**	**< 0.0001**	**< 0.0001**	**< 0.0001**	**< 0.0001**	**< 0.0001**	**< 0.0001**	**< 0.0001**	>0.999	0.473	**< 0.0001**	**< 0.0001**	**< 0.0001**
		pre-ex	**21.3±1.6**	**22.5±12.1**	**1.2±0.7**	**1.7±2.7**	**7.5±4.2**	**14.9±3.8**	**22.61.8**	**26.0±0.0**	26.0±0.0	0.2±0.1	**26.0±0.0**	**1.0±0.1**	**10.0±0.0**
**G**	M	post-ex	**13.9±2.8**	**68.9±7.8**	**0.0±0.5**	**-1.3±0.4**	**3.0±0.0**	**3.2±0.5**	**13.7±5.6**	**25.1±1.3**	26.0±0.0	0.2±0.1	**7.8±9.5**	**0.7±0.1**	**5.7±4.1**
		p	**< 0.0001**	**< 0.0001**	**< 0.0001**	**< 0.0001**	**< 0.0001**	**< 0.0001**	**< 0.0001**	**0.0005**	>0.999	0.768	**< 0.0001**	**< 0.0001**	**< 0.0001**
		pre-ex	**21.6±1.5**	**20.7±8.9**	**1.4±0.7**	**2.6±2.7**	**7.1±4.2**	**15.8±2.9**	**22.8±1.6**	**26.0±0.0**	26.0±0.0	0.2±0.1	**26.0±0.0**	**1.0±0.0**	**10.0±0.0**
	H	post-ex	**13.3±3.2**	**65.1±14.8**	**-0.1±0.6**	**-1.1±0.8**	**3.0±0.2**	**3.4±1.2**	**13.0±5.5**	**24.2±2.6**	25.9±0.3	0.2±0.1	**7.8±9.5**	**0.7±0.1**	**5.7±4.1**
		p	**< 0.0001**	**< 0.0001**	**< 0.0001**	**< 0.0001**	**< 0.0001**	**< 0.0001**	**< 0.0001**	**< 0.0001**	0.500	0.663	**< 0.0001**	**< 0.0001**	**< 0.0001**
		pre-ex	5.4±3.5	**55.5±40.0**	**-1.5±0.8**	1.9±3.3	0.0±0.0	0.0±0.0	**2.5±2.7**	**16.4±9.2**	**21.8±8.3**	**0.4±0.1**	**0.0±0.0**	**0.5±0.1**	1.4±3.7
	L	post-ex	4.2±1.1	**9.1±3.1**	**-0.5±0.8**	0.6±3.2	0.0±0.2	0.5±0.8	**3.9±1.5**	**8.1±1.1**	**11.0±3.0**	**0.2±0.1**	**2.6±3.3**	**0.8±0.1**	0.0±0.0
		p	0.241	**< 0.0001**	**< 0.0001**	0.178	>0.999	0.008	**0.021**	**0.0009**	**< 0.0001**	**< 0.0001**	**0.001**	**< 0.0001**	0.250
		pre-ex	5.4±3.4	**53.5±38.0**	**-1.5±0.8**	1.9±3.2	0.0±0.0	0.0±0.0	**2.7±4.0**	**16.3±8.1**	**22.5±6.2**	**0.4±0.1**	**0.0±0.0**	**0.5±0.1**	1.4±3.7
**B**	M	post-ex	4.1±1.0	**10.1±3.3**	**-0.7±0.8**	1.4±3.9	0.0±0.0	0.3±0.5	**3.8±1.6**	**8.1±0.9**	**11.9±3.9**	**0.2±0.1**	**2.7±3.2**	**0.8±0.1**	0.0±0.0
		p	0.188	**< 0.0001**	**0.007**	0.473	-	0.063	**0.016**	**< 0.0001**	**< 0.0001**	**< 0.0001**	**< 0.0001**	**< 0.0001**	0.250
		pre-ex	5.4±3.4	**55.1±38.6**	**-1.7±1.1**	3.8±6.5	0.0±0.0	0.0±0.0	**2.3±2.9**	**16.0±8.7**	**23.0±6.2**	**0.4±0.1**	**0.0±0.0**	**0.5±0.1**	0.9±3.1
	H	post-ex	4.4±1.1	**11.5±5.8**	**-0.7±0.8**	1.3±3.2	0.0±0.0	0.6±0.9	**3.9±1.4**	**8.6±1.3**	**13.1±5.1**	**0.2±0.1**	**2.4±3.3**	**0.8±0.1**	0.0±0.0
		p	0.375	**< 0.0001**	**0.008**	0.121	-	0.004	**< 0.0001**	**< 0.0001**	**< 0.0001**	**< 0.0001**	**< 0.0001**	**< 0.0001**	0.500

R - Red component; G - Green component; B - Blue component. Skewness - skewness coefficient; Perc01, Perc10, Perc50, Perc90, Perc99 - percentiles; Domn01, Domn10 - dominants; Maxm01, Maxm10 - maximum of moments; p - the level of marginal significance; SD - standard deviation.

Table S9. The withers area (ROI 1). Values (mean ± SD) of Gray Level Run Length Matrix (GLRLM) features for three color components (R, red; G, green; B, blue) in light (L), moderate (M), and heavy (H) groups. The pre-exercise (pre-ex) and post exercise (post-ex) data series were compared. When features differed significantly (p<0.05) for all three groups (L, M, H), the values were marked in bold.

Groups			GLN	RLN	LRE	SRE	Fraction	MRLN	MGLN
		pre-ex	**3999.0±3027.0**	577.0±153.1	123.6±132.0	**0.6±0.1**	**0.3±0.1**	**0.3±0.1**	**0.1±0.0**
	L	post-ex	**881.8±1106.0**	555.0±217.1	1252.0±1382.0	**0.3±0.1**	**0.1±0.1**	**0.1±0.1**	**0.2±0.1**
		p	**< 0.0001**	0.527	< 0.0001	**< 0.0001**	**< 0.0001**	**< 0.0001**	**< 0.0001**
		pre-ex	**4843.0±3631.0**	616.4±198.6	110.8±122.0	**0.6±0.1**	**0.3±0.1**	**0.3±0.1**	**0.1±0.0**
**R**	M	post-ex	**1429.0±1389.0**	589.6±176.3	681.7±740.4	**0.4±0.2**	**0.1±0.1**	**0.2±0.1**	**0.1±0.1**
		p	**< 0.0001**	0.833	< 0.0001	**0.0004**	**< 0.0001**	**< 0.0001**	**< 0.0001**
		pre-ex	**5550.0±3400.0**	637.0±170.1	73.2±91.0	**0.6±0.1**	**0.4±0.1**	**0.4±0.1**	**0.1±0.0**
	H	post-ex	**3343.0±3581.0**	702.4±184.5	256.5±234.2	**0.5±0.2**	**0.2±0.1**	**0.2±0.1**	**0.1±0.0**
		p	**0.014**	0.121	0.070	**0.0004**	**< 0.0001**	**0.0004**	**0.0001**
		pre-ex	**2702.0±1718.0**	821.1±242.3	37.5±18.4	**0.5±0.1**	**0.3±0.1**	**0.2±0.1**	**0.1±0.0**
	L	post-ex	**6227.0±2700.0**	792.2±239.9	29.7±12.8	**0.6±0.1**	**0.4±0.1**	**0.4±0.1**	**0.0±0.0**
		p	**< 0.0001**	0.705	0.241	**< 0.0001**	**< 0.0001**	**< 0.0001**	**< 0.0001**
		pre-ex	**2833.0±1684.0**	905.8±305.3	45.3±27.0	**0.4±0.1**	**0.3±0.1**	**0.2±0.1**	**0.1±0.0**
**G**	M	post-ex	**5817.0±1564.0**	785.8±170.3	29.8±8.8	**0.6±0.0**	**0.4±0.0**	**0.4±0.0**	**0.0±0.0**
		p	**< 0.0001**	0.095	0.105	**< 0.0001**	**< 0.0001**	**< 0.0001**	**< 0.0001**
		pre-ex	**2649.0±1507.0**	943.6±344.6	43.9±27.5	**0.5±0.1**	**0.3±0.1**	**0.2±0.1**	**0.1±0.0**
	H	post-ex	**6094.0±1816.0**	946.0±260.6	31.5±20.0	**0.6±0.0**	**0.4±0.1**	**0.3±0.0**	**0.1±0.0**
		p	**< 0.0001**	0.833	0.150	**< 0.0001**	**< 0.0001**	**< 0.0001**	**< 0.0001**
		pre-ex	1644.0±2066.0	**675.5±209.3**	**192.5±140.4**	**0.4±0.2**	0.2±0.1	0.2±0.1	0.1±0.1
	L	post-ex	918.4±484.6	**867.4±276.5**	**88.5±56.1**	**0.2±0.1**	0.2±0.0	0.1±0.0	0.1±0.0
		p	0.197	**0.013**	**0.004**	**0.0008**	0.989	0.056	0.277
		pre-ex	2114.0±2456.0	**689.3±177.5**	**207.2±164.2**	**0.4±0.2**	0.2±0.1	0.2±0.1	0.1±0.1
**B**	M	post-ex	1039.0±521.6	**832.5±194.6**	**93.2±52.3**	**0.3±0.1**	0.2±0.0	0.1±0.0	0.1±0.0
		p	0.128	**0.007**	**0.0008**	**0.0008**	0.705	0.087	0.197
		pre-ex	2324.0±2496.0	**674.9±195.7**	**217.4±203.7**	**0.4±0.2**	0.2±0.1	0.2±0.1	0.1±0.1
	H	post-ex	1892.0±1834.0	**929.9±216.4**	**118.8±83.0**	**0.3±0.2**	0.2±0.1	0.2±0.1	0.1±0.0
		p	0.899	**< 0.0001**	**0.015**	**0.018**	0.584	0.164	0.406

R - Red component; G - Green component; B - Blue component. GLN - gray level non-uniformity, RLN - run-length nonuniformity, LRE - long-run emphasis, SRE - short-run emphasis, Fraction - a fraction of image in runs, MRLN - run-length nonuniformity moment, MGLN - gray level non-uniformity moment.

Table S10. The thoracic spine area (ROI 2)**.** Values (mean ± SD) of Gray Level Run Length Matrix (GLRLM) features for three color components (R, red; G, green; B, blue) in light (L), moderate (M), and heavy (H) groups. The pre-exercise (pre-ex) and post exercise (post-ex) data series were compared. When features differed significantly (p<0.05) for all three groups (L, M, H), the values were marked in bold.

Groups			GLN	RLN	LRE	SRE	Fraction	MRLN	MGLN
		pre-ex	1139.0±1625.0	**469.2±158.5**	1266.0±1251.0	0.3±0.2	0.1±0.1	0.2±0.1	0.2±0.2
	L	post-ex	588.4±947.0	**644.5±246.9**	1045.0±804.1	0.2±0.1	0.1±0.1	0.1±0.1	0.3±0.1
		p	0.241	**0.003**	0.473	0.016	0.643	0.005	0. 160
		pre-ex	1644.0±1984.0	**491.4±137.2**	963.9±884.3	0.4±0.2	0.1±0.1	0.2±0.1	0.2±0.1
**R**	M	post-ex	1333.0±2075.0	**782.1±409.5**	782.5±925.1	0.3±0.2	0.1±0.1	0.1±0.1	0.2±0.2
		p	0.345	**0.0008**	0.303	0.025	0.584	0.049	0.160
		pre-ex	1725.0±1829.0	**464.8±176.5**	962.1±1222.0	0.4±0.2	0.1±0.1	0.2±0.1	0.1±0.1
	H	post-ex	2209.0±3335.0	**839.5±304.9**	741.3±1129.0	0.3±0.2	0.2±0.1	0.2±0.1	0.2±0.1
		p	0.790	**< 0.0001**	0.360	0.290	0.128	0.060	0.011
		pre-ex	4194.0±2171.0	694.0±234.2	40.7±24.8	0.5±0.1	0.3±0.1	0.3±0.1	**0.0±0.0**
	L	post-ex	5094.0±2277.0	853.5±254.7	20.2±11.6	0.6±0.1	0.4±0.1	0.3±0.1	**0.1±0.0**
		p	0.089	0.014	0.002	0.017	0.007	0.043	**0.034**
		pre-ex	4280.0±2270.0	760.9±233.5	45.6±23.5	0.5±0.1	0.3±0.1	0.3±0.1	**0.0±0.0**
**G**	M	post-ex	4780.0±2100.0	952.2±422.7	29.1±19.1	0.5±0.1	0.3±0.1	0.3±0.1	**0.1±0.0**
		p	0.208	0.102	0.008	0.108	0.160	0.065	**0.0006**
		pre-ex	3993.0±1851.0	780.2±278.2	35.7±23.1	0.5±0.1	0.3±0.1	0.3±0.1	**0.1±0.0**
	H	post-ex	5417.0±2937.0	1084.0±443.3	36.8±38.8	0.5±0.1	0.3±0.1	0.3±0.1	**0.1±0.0**
		p	0.009	0.008	0.491	0.004	0.208	0.015	**0.004**
		pre-ex	602.8±664.1	**732.7±259.1**	132.6±56.9	0.2±0.1	0.1±0.0	0.1±0.0	0.1±0.0
	L	post-ex	582.0±292.3	**915.8±264.2**	123.9±69.3	0.2±0.1	0.1±0.0	0.1±0.0	0.2±0.0
		p	0.345	**0.005**	0.197	0.422	0.491	0.152	0.063
		pre-ex	775.1±953.9	**777.0±208.2**	152.1±83.6	0.2±0.1	0.1±0.0	0.1±0.1	0.1±0.0
**B**	M	post-ex	598.4±370.2	**963.6±385.9**	169.6±137.9	0.2±0.1	0.1±0.0	0.1±0.0	0.2±0.0
		p	0.747	**0.016**	0.406	0.833	0.663	0.345	0.093
		pre-ex	696.0±731.7	**772.2±253.1**	149.7±66.2	0.2±0.1	0.1±0.0	0.1±0.0	0.1±0.0
	H	post-ex	897.3±661.1	**1079.0±435.5**	166.2±82.6	0.2±0.1	0.1±0.0	0.1±0.0	0.1±0.0
		p	0.095	**< 0.0001**	0.422	0.331	0.241	0.095	0.126

R - Red component; G - Green component; B - Blue component. GLN - gray level non-uniformity, RLN - run-length nonuniformity, LRE - long-run emphasis, SRE - short-run emphasis, Fraction - a fraction of image in runs, MRLN - run-length nonuniformity moment, MGLN - gray level non-uniformity moment.

Table S11. The left area of back musculature (ROI 3)**.** Values (mean ± SD) of Gray Level Run Length Matrix (GLRLM) features for three color components (R, red; G, green; B, blue) in light (L), moderate (M), and heavy (H) groups. The pre-exercise (pre-ex) and post exercise (post-ex) data series were compared. When features differed significantly ($p<0.05$) for all three groups (L, M, H), the values were marked in bold.

**Groups**			**GLN**	**RLN**	**LRE**	**SRE**	**Fraction**	**MRLN**	**MGLN**
		pre-ex	**7310.0±3772.0**	881.4±265.3	**121.5±143.5**	**0.6±0.1**	**0.3±0.1**	**0.3±0.1**	**0.0±0.0**
	L	post-ex	**1121.0±1531.0**	957.3±277.1	**1299.0±1191.0**	**0.3±0.2**	**0.1±0.0**	**0.1±0.1**	**0.2±0.1**
		p	**< 0.0001**	0.143	**< 0.0001**	**< 0.0001**	**< 0.0001**	**< 0.0001**	**< 0.0001**
		pre-ex	**8421.0±4264.0**	1000.0±248.4	**76.4±67.8**	**0.6±0.1**	**0.3±0.1**	**0.3±0.1**	**0.0±0.0**
**R**	M	post-ex	**1460.0±1589.0**	874.7±267.1	**762.2±545.4**	**0.3±0.1**	**0.1±0.0**	**0.1±0.1**	**0.1±0.1**
		p	**< 0.0001**	0.022	**< 0.0001**	**< 0.0001**	**< 0.0001**	**< 0.0001**	**< 0.0001**
		pre-ex	**9167.0±4049.0**	980.1±260.5	**78.8±59.7**	**0.6±0.1**	**0.3±0.1**	**0.3±0.1**	**0.0±0.0**
	H	post-ex	**3648.0±4519.0**	1075.0±358.0	**669.8±638.1**	**0.4±0.2**	**0.1±0.1**	**0.2±0.1**	**0.1±0.1**
		p	**< 0.0001**	0.345	**< 0.0001**	**< 0.0001**	**< 0.0001**	**< 0.0001**	**< 0.0001**
		pre-ex	**3193.0±1419.0**	1312.0±388.0	56.3±21.6	**0.4±0.1**	**0.2±0.0**	**0.2±0.0**	**0.1±0.0**
	L	post-ex	**7833.0±2987.0**	1260.0±314.7	60.0±24.5	**0.6±0.1**	**0.3±0.0**	**0.3±0.1**	**0.0±0.0**
		p	**< 0.0001**	0.527	< 0.0001	**< 0.0001**	**< 0.0001**	**< 0.0001**	**< 0.0001**
		pre-ex	**3156.0±1685.0**	1467.0±398.9	72.7±40.2	**0.3±0.1**	**0.2±0.0**	**0.2±0.0**	**0.1±0.0**
**G**	M	post-ex	**7720.0±3095.0**	1180.0±340.2	63.9±26.7	**0.5±0.1**	**0.3±0.0**	**0.3±0.0**	**0.0±0.0**
		p	**< 0.0001**	0.002	< 0.0001	**< 0.0001**	**< 0.0001**	**< 0.0001**	**< 0.0001**
		pre-ex	**3283.0±1598.0**	1417.0±417.0	69.6±39.7	**0.4±0.1**	**0.2±0.0**	**0.2±0.0**	**0.1±0.0**
	H	post-ex	**9175.0±3906.0**	1443.0±516.6	55.7±29.1	**0.6±0.1**	**0.3±0.1**	**0.3±0.0**	**0.0±0.0**
		p	**< 0.0001**	0.422	0.107	**< 0.0001**	**< 0.0001**	**< 0.0001**	**< 0.0001**
		pre-ex	**4863.0±3582.0**	1007.0±295.9	174.5±163.8	**0.5±0.2**	**0.2±0.1**	**0.2±0.1**	**0.1±0.1**
	L	post-ex	**1306.0±693.6**	1348.0±368.5	136.2±63.8	**0.2±0.1**	**0.1±0.0**	**0.1±0.0**	**0.1±0.0**
		p	**< 0.0001**	0.125	0.663	**< 0.0001**	**0.004**	**< 0.0001**	**0.002**
		pre-ex	**4988.0±3765.0**	1083.0±280.7	236.7±225.3	**0.5±0.1**	**0.2±0.1**	**0.2±0.1**	**0.1±0.0**
**B**	M	post-ex	**1293.0±787.8**	1269.0±384.4	169.1±70.3	**0.2±0.1**	**0.1±0.0**	**0.1±0.0**	**0.1±0.0**
		p	**< 0.0001**	0.065	0.500	**< 0.0001**	**< 0.0001**	**< 0.0001**	**< 0.0001**
		pre-ex	**5636.0±4280.0**	1054.0±300.0	235.9±250.7	**0.5±0.2**	**0.2±0.1**	**0.3±0.1**	**0.1±0.1**
	H	post-ex	**2094.0±1954.0**	1485.0±489.1	175.5±141.2	**0.2±0.1**	**0.2±0.0**	**0.1±0.0**	**0.1±0.0**
		p	**0.004**	0.0004	0.406	**< 0.0001**	**0.003**	**< 0.0001**	**0.0004**

R - Red component; G - Green component; B - Blue component. GLN - gray level non-uniformity, RLN - run-length nonuniformity, LRE - long-run emphasis, SRE - short-run emphasis, Fraction - a fraction of image in runs, MRLN - run-length nonuniformity moment, MGLN - gray level non-uniformity moment.

Table S12. The right area of back musculature (ROI 4)**.** Values (mean ± SD) of Gray Level Run Length Matrix (GLRLM) features for three color components (R, red; G, green; B, blue) in light (L), moderate (M), and heavy (H) groups. The pre-exercise (pre-ex) and post exercise (post-ex) data series were compared. When features differed significantly (p<0.05) for all three groups (L, M, H), the values were marked in bold.

Groups			GLN	RLN	LRE	SRE	Fraction	MRLN	MGLN
R	L	pre-ex	**6726.0±4201.0**	818.0±276.6	**87.8±58.0**	**0.5±0.1**	**0.3±0.1**	**0.3±0.1**	**0.0±0.0**
		post-ex	**1307.0±2158.0**	773.8±243.8	**958.4±814.0**	**0.3±0.1**	**0.1±0.1**	**0.1±0.1**	**0.1±0.1**
		p	**< 0.0001**	0.989	**< 0.0001**	**< 0.0001**	**< 0.0001**	**< 0.0001**	**< 0.0001**
	M	pre-ex	**7729.0±3900.0**	875.4±176.5	**98.3±124.7**	**0.6±0.1**	**0.3±0.1**	**0.3±0.1**	**0.0±0.0**
		post-ex	**1389.0±1213.0**	759.0±288.3	**731.3±458.9**	**0.4±0.1**	**0.1±0.0**	**0.1±0.1**	**0.1±0.1**
		p	**< 0.0001**	0.065	**< 0.0001**	**< 0.0001**	**< 0.0001**	**< 0.0001**	**< 0.0001**
	H	pre-ex	**7908.0±3006.0**	836.8±232.6	**53.6±31.4**	**0.6±0.1**	**0.3±0.1**	**0.3±0.1**	**0.0±0.0**
		post-ex	**2177.0±3156.0**	836.8±366.4	**911.9±1121.0**	**0.4±0.2**	**0.1±0.1**	**0.2±0.1**	**0.1±0.1**
		p	**< 0.0001**	0.456	**< 0.0001**	**< 0.0001**	**< 0.0001**	**< 0.0001**	**< 0.0001**
G	L	pre-ex	**2750.0±1808.0**	1180.0±397.3	73.0±56.8	**0.3±0.1**	**0.2±0.1**	**0.2±0.0**	**0.1±0.0**
		post-ex	**7344.0±3204.0**	1121.0±361.1	56.9±28.4	**0.6±0.1**	**0.3±0.1**	**0.3±0.1**	**0.1±0.0**
		p	**< 0.0001**	0.456	0.208	**< 0.0001**	**< 0.0001**	**< 0.0001**	**< 0.0001**
	M	pre-ex	**2797.0±1457.0**	1279.0±320.8	68.8±35.3	**0.3±0.1**	**0.2±0.0**	**0.2±0.0**	**0.1±0.0**
		post-ex	**7198.0±2698.0**	1099.0±298.3	56.2±28.3	**0.6±0.1**	**0.3±0.0**	**0.3±0.0**	**0.0±0.0**
		p	**< 0.0001**	0.013	0.500	**< 0.0001**	**< 0.0001**	**< 0.0001**	**< 0.0001**
	H	pre-ex	**2438.0±1288.0**	1216.0±385.6	70.9±45.7	**0.3±0.1**	**0.2±0.0**	**0.2±0.0**	**0.1±0.0**
		post-ex	**7663.0±3126.0**	1204.0±408.1	51.4±27.7	**0.6±0.0**	**0.3±0.1**	**0.3±0.0**	**0.0±0.0**
		p	**< 0.0001**	0.491	0.074	**< 0.0001**	**< 0.0001**	**< 0.0001**	**< 0.0001**
B	L	pre-ex	**4438.0±3238.0**	886.8±313.4	276.3±447.4	**0.5±0.2**	**0.2±0.1**	**0.2±0.1**	**0.1±0.1**
		post-ex	**1235.0±916.4**	1206.0±377.2	154.6±101.4	**0.2±0.1**	**0.1±0.0**	**0.1±0.0**	**0.1±0.0**
		p	**< 0.0001**	0.002	0.899	**< 0.0001**	**< 0.0001**	**< 0.0001**	**< 0.0001**
	M	pre-ex	**5330.0±3686.0**	971.3±241.1	179.1±206.4	**0.5±0.2**	**0.2±0.1**	**0.3±0.1**	**0.1±0.0**
		post-ex	**1182.0±643.4**	1171.0±351.4	147.5±81.0	**0.2±0.1**	**0.1±0.0**	**0.1±0.0**	**0.1±0.0**
		p	**< 0.0001**	0.065	0.406	**< 0.0001**	**< 0.0001**	**< 0.0001**	**< 0.0001**
	H	pre-ex	**5218.0±3557.0**	880.7±259.2	200.9±259.0	**0.5±0.1**	**0.2±0.1**	**0.3±0.1**	**0.1±0.0**
		post-ex	**1606.0±1458.0**	1247.0±408.0	121.0±63.7	**0.2±0.1**	**0.2±0.0**	**0.1±0.0**	**0.1±0.0**
		p	**< 0.0001**	0.0006	0.527	**< 0.0001**	**0.001**	**< 0.0001**	**< 0.0001**

R - Red component; G - Green component; B - Blue component. GLN - gray level non-uniformity, RLN - run-length nonuniformity, LRE - long-run emphasis, SRE - short-run emphasis, Fraction - a fraction of image in runs, MRLN - run-length nonuniformity moment, MGLN - gray level non-uniformity moment.

Table S13. The withers area (ROI 1). Values (mean ± SD) of Gray Level Co-occurrence Matrix (GLCM) features for three color components (R, red; G, green; B, blue) in light (L), moderate (M), and heavy (H) groups. The pre-exercise (pre-ex) and post exercise (post-ex) data series were compared. When features differed significantly (p<0.05) for all three groups (L, M, H), the values were marked in bold.

Groups			AngScMom	Contrast	Correlat	SumOfSqs	InvDfMom	SumAverg	SumVarnc	SumEntrp	Entropy	DifVarnc	DifEntrp
		pre-ex	**0.1±0.1**	**0.6±0.4**	1.0±0.0	**48.7±38.3**	**0.8±0.1**	**52.2±8.6**	**194.0±153.0**	**1.3±0.3**	**1.4±0.4**	**0.4±0.3**	**0.3±0.1**
	L	post-ex	**0.5±0.1**	**0.2±0.2**	1.0±0.0	**8.5±10.0**	**0.9±0.0**	**61.9±1.6**	**33.7±39.8**	**0.6±0.2**	**0.6±0.3**	**0.1±0.1**	**0.1±0.1**
		p	**< 0.0001**	**< 0.0001**	< 0.0001	**< 0.0001**	**< 0.0001**	**< 0.0001**	**< 0.0001**	**< 0.0001**	**< 0.0001**	**< 0.0001**	**< 0.0001**
		pre-ex	**0.1±0.1**	**0.7±0.5**	1.0±0.0	**56.9±41.5**	**0.8±0.1**	**50.9±8.3**	**226.8±165.6**	**1.3±0.3**	**1.5±0.4**	**0.5±0.3**	**0.3±0.1**
**R**	M	post-ex	**0.4±0.1**	**0.2±0.2**	1.0±0.0	**15.5±17.7**	**0.9±0.0**	**60.4±3.0**	**61.8±70.7**	**0.7±0.3**	**0.8±0.3**	**0.2±0.2**	**0.2±0.1**
		p	**< 0.0001**	**< 0.0001**	0.0004	**< 0.0001**	**< 0.0001**	**< 0.0001**	**< 0.0001**	**< 0.0001**	**< 0.0001**	**0.0003**	**< 0.0001**
		pre-ex	**0.1±0.1**	**0.8±0.4**	1.0±0.0	**62.8±39.2**	**0.8±0.1**	**49.0±7.4**	**250.6±156.4**	**1.4±0.2**	**1.6±0.3**	**0.5±0.2**	**0.4±0.1**
	H	post-ex	**0.2±0.1**	**0.4±0.4**	1.0±0.0	**33.3±37.7**	**0.9±0.1**	**57.2±6.0**	**132.6±150.6**	**1.0±0.3**	**1.1±0.4**	**0.3±0.3**	**0.3±0.1**
		p	**< 0.0001**	**< 0.0001**	0.151	**0.007**	**< 0.0001**	**< 0.0001**	**0.007**	**< 0.0001**	**< 0.0001**	**0.0006**	**< 0.0001**
		pre-ex	**0.1±0.0**	**0.4±0.2**	**1.0±0.0**	**25.3±16.9**	**0.8±0.0**	**45.1±3.9**	**100.6±67.6**	**1.3±0.2**	**1.4±0.2**	**0.3±0.1**	**0.3±0.1**
	L	post-ex	**0.1±0.0**	**0.7±0.2**	**1.0±0.0**	**61.1±14.2**	**0.8±0.0**	**29.3±6.0**	**243.5±56.5**	**1.5±0.1**	**1.7±0.1**	**0.5±0.1**	**0.4±0.0**
		p	**0.005**	**< 0.0001**	**< 0.0001**	**< 0.0001**	**< 0.0001**	**< 0.0001**	**< 0.0001**	**< 0.0001**	**< 0.0001**	**< 0.0001**	**< 0.0001**
		pre-ex	**0.1±0.1**	**0.4±0.2**	**1.0±0.0**	**23.1±15.5**	**0.8±0.0**	**45.6±3.7**	**92.2±61.8**	**1.3±0.2**	**1.4±0.2**	**0.3±0.1**	**0.3±0.1**
**G**	M	post-ex	**0.1±0.0**	**0.7±0.2**	**1.0±0.0**	**62.5±12.5**	**0.8±0.0**	**31.3±7.2**	**249.1±50.0**	**1.5±0.1**	**1.6±0.1**	**0.5±0.1**	**0.4±0.0**
		p	**0.004**	**< 0.0001**	**< 0.0001**	**< 0.0001**	**< 0.0001**	**< 0.0001**	**< 0.0001**	**< 0.0001**	**< 0.0001**	**< 0.0001**	**< 0.0001**
		pre-ex	**0.1±0.0**	**0.4±0.2**	**1.0±0.0**	**21.3±12.4**	**0.9±0.0**	**46.4±2.5**	**84.9±49.4**	**1.2±0.1**	**1.3±0.2**	**0.3±0.1**	**0.3±0.0**
	H	post-ex	**0.1±0.0**	**0.7±0.2**	**1.0±0.0**	**61.5±17.2**	**0.8±0.0**	**37.1±5.1**	**245.3±68.7**	**1.4±0.1**	**1.6±0.1**	**0.5±0.1**	**0.4±0.0**
		p	**< 0.0001**	**< 0.0001**	**< 0.0001**	**< 0.0001**	**< 0.0001**	**< 0.0001**	**< 0.0001**	**< 0.0001**	**< 0.0001**	**< 0.0001**	**< 0.0001**
		pre-ex	**0.2±0.1**	0.4±0.3	1.0±0.0	16.4±20.2	0.9±0.1	**6.8±3.2**	65.3±80.5	**0.9±0.2**	**1.0±0.3**	0.3±0.2	0.2±0.1
	L	post-ex	**0.1±0.0**	0.2±0.1	1.0±0.0	8.2±2.7	0.9±0.0	**10.1±1.9**	32.5±10.9	**1.2±0.1**	**1.2±0.1**	0.2±0.1	0.2±0.0
		p	**< 0.0001**	< 0.0001	0.394	0.197	0.509	**< 0.0001**	0.197	**< 0.0001**	**0.003**	0.095	0.197
		pre-ex	**0.2±0.1**	0.4±0.5	1.0±0.0	19.5±24.8	0.9±0.1	**7.1±3.7**	77.5±98.9	**0.9±0.2**	**1.0±0.3**	0.3±0.3	0.2±0.1
**B**	M	post-ex	**0.1±0.0**	0.2±0.1	1.0±0.0	9.9±3.9	0.9±0.0	**9.8±2.4**	39.3±15.7	**1.2±0.1**	**1.2±0.1**	0.2±0.1	0.2±0.0
		p	**< 0.0001**	0.107	0.194	0.143	0.989	**0.0008**	0.143	**0.0001**	**0.004**	0.056	0.456
		pre-ex	**0.3±0.1**	0.5±0.4	1.0±0.0	22.2±22.5	0.9±0.1	**7.1±3.4**	88.1±89.5	**0.9±0.2**	**1.0±0.3**	0.4±0.3	0.3±0.1
	H	post-ex	**0.1±0.0**	0.3±0.3	1.0±0.0	14.8±12.3	0.9±0.0	**8.5±1.8**	58.7±49.1	**1.1±0.1**	**1.2±0.1**	0.3±0.2	0.2±0.1
		p	**< 0.0001**	0.105	0.705	0.375	0.944	**0.0009**	0.375	**0.0002**	**0.005**	0.054	0.527

R - Red component; G - Green component; B - Blue component. AngScMom - angular second moment/energy; Correlat - correlation; SumOfSqs - sum of squares; InvDefMom - inverse different moment/homogeneity; SumAverg - summation mean; SumVarnc - summation variance; SumEntrp - summation entropy; DifVarnc - differential variance; DifEntrp - differential entropy; p - the level of marginal significance; SD - standard deviation.

Table S14. The thoracic spine area (ROI 2**).** Values (mean ± SD) of Gray Level Co-occurrence Matrix (GLCM) features for three color components (R, red; G, green; B, blue) in light (L), moderate (M), and heavy (H) groups. The pre-exercise (pre-ex) and post exercise (post-ex) data series were compared. When features differed significantly (p<0.05) for all three groups (L, M, H), the values were marked in bold.

	Groups		AngScMom	Contrast	Correlat	SumOfSqs	InvDfMom	SumAverg	SumVarnc	SumEntrp	Entropy	DifVarnc	DifEntrp
		pre-ex	0.6±0.2	0.2±0.2	1.0±0.0	12.5±20.9	1.0±0.0	61.4±3.4	49.8±83.2	0.5±0.3	0.6±0.4	0.1±0.2	0.1±0.1
	L	post-ex	0.6±0.2	0.1±0.1	1.0±0.0	3.3±4.9	1.0±0.0	62.5±1.6	13.3±19.5	0.5±0.3	0.5±0.3	0.1±0.1	0.1±0.1
		p	>0.999	0.375	0.043	0.049	0.128	0.360	0.049	>0.999	0.944	0.375	0.406
		pre-ex	0.5±0.2	0.2±0.3	1.0±0.0	19.2±28.7	0.9±0.0	60.5±3.7	76.4±114.5	0.6±0.3	0.7±0.4	0.2±0.2	0.2±0.1
**R**	M	post-ex	0.4±0.2	0.2±0.2	1.0±0.0	7.0±9.1	0.9±0.1	61.2±2.9	28.0±36.4	0.7±0.4	0.7±0.4	0.1±0.1	0.2±0.1
		p	0.422	0.375	0.264	0.039	0.747	0.527	0.049	0.643	0.663	0.331	0.504
		pre-ex	0.5±0.2	0.2±0.2	1.0±0.0	18.5±26.0	0.9±0.0	60.5±3.5	73.8±104.0	0.7±0.3	0.7±0.4	0.2±0.2	0.2±0.1
	H	post-ex	0.4±0.3	0.2±0.3	1.0±0.0	12.4±16.9	0.9±0.1	60.2±3.9	49.3±67.3	0.8±0.4	0.8±0.5	0.2±0.2	0.2±0.1
		p	0.107	0.989	0.056	0.277	0.473	0.922	0.277	0.290	0.750	0.944	0.584
		pre-ex	0.1±0.0	**0.4±0.2**	**1.0±0.0**	**57.0±13.7**	0.8±0.0	**29.6±6.0**	**227.5±55.0**	1.5±0.1	1.6±0.1	**0.3±0.1**	**0.3±0.0**
	L	post-ex	0.0±0.0	**0.6±0.2**	**1.0±0.0**	**38.7±10.1**	0.8±0.0	**36.0±6.4**	**154.3±40.3**	1.5±0.1	1.7±0.1	**0.4±0.1**	**0.4±0.0**
		p	0.094	**0.002**	**< 0.0001**	**< 0.0001**	0.004	**0.0003**	**< 0.0001**	0.345	0.101	**0.0008**	**0.002**
		pre-ex	0.1±0.0	**0.4±0.2**	**1.0±0.0**	**56.9±15.8**	0.8±0.0	**31.4±6.5**	**227.4±63.4**	1.5±0.1	1.6±0.1	**0.3±0.1**	**0.3±0.1**
**G**	M	post-ex	0.1±0.1	**0.5±0.2**	**1.0±0.0**	**38.5±14.8**	0.8±0.0	**38.0±7.9**	**153.3±59.3**	1.4±0.2	1.6±0.2	**0.4±0.1**	**0.3±0.0**
		p	0.726	**0.004**	**< 0.0001**	**< 0.0001**	0.079	**< 0.0001**	**< 0.0001**	0.264	0.726	**0.0006**	**0.005**
		pre-ex	0.1±0.0	**0.4±0.1**	**1.0±0.0**	**49.8±15.8**	0.8±0.0	**33.3±7.4**	**198.6±63.1**	1.5±0.1	1.6±0.1	**0.3±0.1**	**0.3±0.0**
	H	post-ex	0.1±0.1	**0.5±0.2**	**1.0±0.0**	**35.2±11.8**	0.8±0.0	**39.1±8.3**	**140.3±47.1**	1.4±0.2	1.6±0.2	**0.4±0.1**	**0.3±0.1**
		p	0.053	**0.004**	**< 0.0001**	**< 0.0001**	0.084	**< 0.0001**	**< 0.0001**	0.019	0.264	**0.001**	**0.006**
		pre-ex	**0.1±0.0**	0.2±0.2	**1.0±0.0**	**6.9±4.5**	0.9±0.0	**9.6±1.7**	**27.3±17.8**	**1.1±0.1**	**1.1±0.1**	0.1±0.1	0.2±0.0
	L	post-ex	**0.1±0.1**	0.1±0.0	**1.0±0.0**	**4.0±1.5**	0.9±0.0	**7.6±1.9**	**15.8±6.2**	**1.0±0.1**	**1.0±0.1**	0.1±0.0	0.2±0.0
		p	**0.004**	0.290	**< 0.0001**	**< 0.0001**	0.169	**0.0003**	**< 0.0001**	**0.010**	**0.013**	0.360	0.208
		pre-ex	**0.1±0.0**	0.2±0.2	**1.0±0.0**	**9.2±9.9**	0.9±0.0	**9.4±2.0**	**36.7±39.2**	**1.1±0.1**	**1.1±0.1**	0.2±0.2	0.2±0.1
**B**	M	post-ex	**0.2±0.1**	0.1±0.0	**1.0±0.0**	**4.1±1.8**	0.9±0.0	**7.0±2.4**	**16.5±7.0**	**0.9±0.1**	**1.0±0.1**	0.1±0.0	0.2±0.0
		p	**< 0.0001**	0.160	**0.0003**	**< 0.0001**	0.944	**< 0.0001**	**< 0.0001**	**< 0.0001**	**< 0.0001**	0.623	0.197
		pre-ex	**0.1±0.0**	0.2±0.2	**1.0±0.0**	**8.2±8.8**	0.9±0.0	**8.7±2.3**	**32.6±34.9**	**1.0±0.1**	**1.1±0.1**	0.2±0.1	0.2±0.0
	H	post-ex	**0.2±0.1**	0.2±0.1	**1.0±0.0**	**5.0±3.4**	0.9±0.0	**6.8±2.5**	**19.9±13.6**	**0.9±0.1**	**1.0±0.1**	0.2±0.1	0.2±0.0
		p	**0.0003**	0.726	**0.003**	**0.016**	0.208	**0.003**	**0.018**	**0.001**	**0.002**	0.833	0.375

R - Red component; G - Green component; B - Blue component. AngScMom - angular second moment/energy; Correlat - correlation; SumOfSqs - sum of squares; InvDefMom - inverse different moment/homogeneity; SumAverg - summation mean; SumVarnc - summation variance; SumEntrp - summation entropy; DifVarnc - differential variance; DifEntrp - differential entropy; p - the level of marginal significance; SD - standard deviation.

Table S15. The left area of back musculature (ROI 3)**.** Values (mean ± SD) of Gray Level Co-occurrence Matrix (GLCM) features for three color components (R, red; G, green; B, blue) in light (L), moderate (M), and heavy (H) groups. The pre-exercise (pre-ex) and post exercise (post-ex) data series were compared. When features differed significantly ($p<0.05$) for all three groups (L, M, H), the values were marked in bold.

Groups			AngScMom	Contrast	Correlat	SumOfSqs	InvDfMom	SumAverg	SumVarnc	SumEntrp	Entropy	DifVarnc	DifEntrp
		pre-ex	**0.1±0.1**	**0.5±0.2**	**1.0±0.0**	**101.8±51.6**	**0.8±0.0**	**41.2±11.9**	**406.7±206.4**	**1.4±0.2**	**1.5±0.3**	**0.3±0.1**	**0.3±0.1**
	L	post-ex	**0.4±0.1**	**0.1±0.1**	**1.0±0.0**	**11.1±16.0**	**1.0±0.0**	**61.4±2.3**	**44.3±64.1**	**0.6±0.2**	**0.6±0.3**	**0.1±0.1**	**0.1±0.1**
		p	**< 0.0001**	**< 0.0001**	**< 0.0001**	**< 0.0001**	**< 0.0001**	**< 0.0001**	**< 0.0001**	**< 0.0001**	**< 0.0001**	**< 0.0001**	**< 0.0001**
		pre-ex	**0.1±0.0**	**0.5±0.2**	**1.0±0.0**	**96.5±42.3**	**0.8±0.0**	**42.1±9.4**	**385.7±169.0**	**1.5±0.2**	**1.6±0.2**	**0.3±0.2**	**0.3±0.1**
**R**	M	post-ex	**0.4±0.1**	**0.1±0.1**	**1.0±0.0**	**12.8±16.1**	**1.0±0.0**	**61.0±2.0**	**51.0±64.2**	**0.7±0.2**	**0.7±0.2**	**0.1±0.1**	**0.1±0.1**
		p	**< 0.0001**	**< 0.0001**	**< 0.0001**	**< 0.0001**	**< 0.0001**	**< 0.0001**	**< 0.0001**	**< 0.0001**	**< 0.0001**	**< 0.0001**	**< 0.0001**
		pre-ex	**0.1±0.0**	**0.5±0.2**	**1.0±0.0**	**95.6±41.4**	**0.8±0.0**	**41.0±11.1**	**381.9±165.4**	**1.5±0.1**	**1.6±0.2**	**0.4±0.2**	**0.3±0.1**
	H	post-ex	**0.3±0.1**	**0.2±0.2**	**1.0±0.0**	**25.0±31.6**	**0.9±0.1**	**59.2±3.9**	**99.6±126.1**	**0.8±0.3**	**0.9±0.3**	**0.2±0.2**	**0.2±0.1**
		p	**< 0.0001**	**< 0.0001**	**0.002**	**< 0.0001**	**< 0.0001**	**< 0.0001**	**< 0.0001**	**< 0.0001**	**< 0.0001**	**< 0.0001**	**< 0.0001**
		pre-ex	0.1±0.0	**0.3±0.1**	**1.0±0.0**	**26.0±11.1**	**0.9±0.0**	**43.9±2.9**	**103.9±44.4**	**1.3±0.1**	**1.4±0.1**	**0.2±0.1**	**0.3±0.0**
	L	post-ex	0.1±0.0	**0.5±0.1**	**1.0±0.0**	**62.4±13.9**	**0.8±0.0**	**27.2±6.9**	**248.9±55.6**	**1.4±0.1**	**1.5±0.1**	**0.3±0.1**	**0.3±0.0**
		p	0.406	**< 0.0001**	**0.001**	**< 0.0001**	**< 0.0001**	**< 0.0001**	**< 0.0001**	**0.004**	**0.0004**	**< 0.0001**	**< 0.0001**
		pre-ex	0.1±0.0	**0.3±0.1**	**1.0±0.0**	**22.4±9.7**	**0.9±0.0**	**45.3±2.9**	**89.2±38.8**	**1.3±0.1**	**1.4±0.2**	**0.2±0.1**	**0.2±0.0**
**G**	M	post-ex	0.1±0.0	**0.5±0.1**	**1.0±0.0**	**73.4±8.8**	**0.8±0.0**	**30.6±4.4**	**293.1±35.3**	**1.4±0.1**	**1.6±0.1**	**0.3±0.1**	**0.3±0.0**
		p	0.020	**< 0.0001**	**< 0.0001**	**< 0.0001**	**< 0.0001**	**< 0.0001**	**< 0.0001**	**< 0.0001**	**< 0.0001**	**< 0.0001**	**< 0.0001**
		pre-ex	0.1±0.0	**0.3±0.1**	**1.0±0.0**	**23.8±9.2**	**0.9±0.0**	**44.8±3.1**	**94.9±36.9**	**1.3±0.1**	**1.4±0.1**	**0.2±0.1**	**0.2±0.0**
	H	post-ex	0.1±0.0	**0.5±0.1**	**1.0±0.0**	**63.7±15.7**	**0.8±0.0**	**31.8±7.6**	**254.4±62.9**	**1.4±0.1**	**1.6±0.1**	**0.4±0.1**	**0.3±0.0**
		p	0.197	**< 0.0001**	**< 0.0001**	**< 0.0001**	**< 0.0001**	**< 0.0001**	**< 0.0001**	**< 0.0001**	**< 0.0001**	**< 0.0001**	**< 0.0001**
		pre-ex	**0.2±0.1**	**0.4±0.2**	1.0±0.0	58.7±43.6	0.9±0.1	13.4±7.5	**234.5±174.4**	1.2±0.3	1.3±0.3	**0.3±0.2**	**0.3±0.1**
	L	post-ex	**0.1±0.0**	**0.2±0.0**	1.0±0.0	**9.1±2.6**	**0.9±0.0**	11.1±2.5	**36.3±10.5**	1.2±0.1	1.2±0.1	**0.1±0.0**	**0.2±0.0**
		p	**< 0.0001**	**< 0.0001**	0.004	**0.001**	**0.0004**	0.500	**< 0.0001**	0.241	0.121	**0.0004**	**0.010**
		pre-ex	**0.2±0.1**	**0.4±0.3**	1.0±0.0	**45.6±37.4**	**0.9±0.1**	11.1±6.3	**182.2±149.3**	1.1±0.3	1.2±0.3	**0.3±0.2**	**0.3±0.1**
**B**	M	post-ex	**0.1±0.0**	**0.2±0.1**	1.0±0.0	**10.2±3.0**	**0.9±0.0**	9.8±1.3	**40.8±11.9**	1.1±0.0	1.2±0.1	**0.1±0.1**	**0.2±0.0**
		p	**< 0.0001**	**< 0.0001**	0.126	**< 0.0001**	**< 0.0001**	0.422	**< 0.0001**	0.623	>0.999	**0.0002**	**< 0.0001**
		pre-ex	**0.2±0.1**	**0.4±0.3**	1.0±0.0	**52.0±43.0**	**0.9±0.1**	12.4±7.8	**207.6±171.6**	1.1±0.3	1.2±0.3	**0.3±0.2**	**0.3±0.1**
	H	post-ex	**0.1±0.0**	**0.2±0.1**	1.0±0.0	**12.7±9.8**	**0.9±0.0**	9.9±2.6	**50.4±39.1**	1.1±0.1	1.2±0.1	**0.2±0.1**	**0.2±0.1**
		p	**0.0004**	**0.020**	0.094	**< 0.0001**	**0.001**	0.833	**0.001**	0.979	0.491	**0.0008**	**0.004**

R - Red component; G - Green component; B - Blue component. AngScMom - angular second moment/energy; Correlat - correlation; SumOfSqs - sum of squares; InvDefMom - inverse different moment/homogeneity; SumAverg - summation mean; SumVarnc - summation variance; SumEntrp - summation entropy; DifVarnc - differential variance; DifEntrp - differential entropy; p - the level of marginal significance; SD - standard deviation.

Table S16. The right area of back musculature (ROI 4). Values (mean ± SD) of Gray Level Co-occurrence Matrix (GLCM) features for three color components (R, red; G, green; B, blue) in light (L), moderate (M), and heavy (H) groups. The pre-exercise (pre-ex) and post exercise (post-ex) data series were compared. When features differed significantly (p<0.05) for all three groups (L, M, H), the values were marked in bold.

Groups			AngScMom	Contrast	Correlat	SumOfSqs	InvDfMom	SumAverg	SumVarnc	SumEntrp	Entropy	DifVarnc	DifEntrp
		pre-ex	**0.1±0.1**	**0.5±0.2**	**1.0±0.0**	**100.0±47.6**	**0.8±0.1**	**40.9±11.2**	**399.3±190.2**	**1.4±0.2**	**1.6±0.3**	**0.3±0.2**	**0.3±0.1**
	L	post-ex	**0.4±0.1**	**0.1±0.2**	**1.0±0.0**	**11.1±16.5**	**1.0±0.0**	**61.3±2.6**	**44.2±65.7**	**0.6±0.2**	**0.7±0.3**	**0.1±0.1**	**0.1±0.1**
		p	**< 0.0001**	**< 0.0001**	**< 0.0001**	**< 0.0001**	**< 0.0001**	**< 0.0001**	**< 0.0001**	**< 0.0001**	**< 0.0001**	**< 0.0001**	**< 0.0001**
		pre-ex	**0.1±0.1**	**0.5±0.2**	**1.0±0.0**	**100.4±39.9**	**0.8±0.1**	**39.3±10.5**	**401.2±159.4**	**1.5±0.2**	**1.6±0.3**	**0.4±0.1**	**0.3±0.1**
**R**	M	post-ex	**0.4±0.1**	**0.1±0.1**	**1.0±0.0**	**15.4±13.9**	**0.9±0.0**	**60.8±1.9**	**61.5±55.3**	**0.7±0.2**	**0.7±0.2**	**0.1±0.1**	**0.1±0.1**
		p	**< 0.0001**	**< 0.0001**	**< 0.0001**	**< 0.0001**	**< 0.0001**	**< 0.0001**	**< 0.0001**	**< 0.0001**	**< 0.0001**	**< 0.0001**	**< 0.0001**
		pre-ex	**0.1±0.0**	**0.6±0.2**	**1.0±0.0**	**102.0±39.2**	**0.8±0.0**	**38.6±9.2**	**407.5±156.7**	**1.5±0.1**	**1.7±0.2**	**0.4±0.1**	**0.3±0.1**
	H	post-ex	**0.4±0.1**	**0.2±0.2**	**1.0±0.0**	**19.7±25.2**	**0.9±0.0**	**60.3±3.2**	**78.8±100.7**	**0.7±0.3**	**0.8±0.3**	**0.2±0.1**	**0.2±0.1**
		p	**< 0.0001**	**< 0.0001**	**< 0.0001**	**< 0.0001**	**< 0.0001**	**< 0.0001**	**< 0.0001**	**< 0.0001**	**< 0.0001**	**< 0.0001**	**< 0.0001**
		pre-ex	0.1±0.1	**0.3±0.1**	**1.0±0.0**	**24.7±11.5**	**0.9±0.0**	**44.3±3.1**	**98.7±46.1**	**1.3±0.2**	**1.4±0.2**	**0.2±0.1**	**0.2±0.0**
	L	post-ex	0.1±0.0	**0.5±0.2**	**1.0±0.0**	**64.5±12.4**	**0.8±0.0**	**28.8±6.6**	**257.5±49.5**	**1.4±0.1**	**1.6±0.1**	**0.4±0.1**	**0.3±0.0**
		p	0.527	**< 0.0001**	**0.004**	**< 0.0001**	**< 0.0001**	**< 0.0001**	**< 0.0001**	**0.0008**	**< 0.0001**	**< 0.0001**	**< 0.0001**
		pre-ex	0.1±0.0	**0.3±0.1**	**1.0±0.0**	**22.4±12.1**	**0.9±0.0**	**44.6±3.1**	**89.5±48.4**	**1.3±0.1**	**1.4±0.1**	**0.2±0.1**	**0.2±0.0**
**G**	M	post-ex	0.1±0.0	**0.5±0.1**	**1.0±0.0**	**68.8±7.8**	**0.8±0.0**	**29.7±5.6**	**274.9±31.2**	**1.4±0.1**	**1.6±0.1**	**0.3±0.1**	**0.3±0.0**
		p	0.500	**< 0.0001**	**< 0.0001**	**< 0.0001**	**< 0.0001**	**< 0.0001**	**< 0.0001**	**< 0.0001**	**< 0.0001**	**< 0.0001**	**< 0.0001**
		pre-ex	0.1±0.1	**0.2±0.1**	**1.0±0.0**	**20.6±8.9**	**0.9±0.0**	**45.3±3.0**	**82.0±35.5**	**1.3±0.2**	**1.3±0.2**	**0.2±0.1**	**0.2±0.0**
	H	post-ex	0.1±0.0	**0.5±0.1**	**1.0±0.0**	**65.1±14.9**	**0.8±0.0**	**28.6±6.5**	**259.8±59.8**	**1.4±0.1**	**1.6±0.1**	**0.4±0.1**	**0.3±0.0**
		p	0.107	**< 0.0001**	**0.0006**	**< 0.0001**	**< 0.0001**	**< 0.0001**	**< 0.0001**	**< 0.0001**	**< 0.0001**	**< 0.0001**	**< 0.0001**
		pre-ex	**0.2±0.1**	**0.4±0.3**	**1.0±0.0**	**55.3±40.0**	**0.9±0.1**	12.9±7.1	**220.6±159.8**	1.1±0.3	1.3±0.3	**0.3±0.2**	**0.3±0.1**
	L	post-ex	**0.1±0.0**	**0.2±0.1**	**1.0±0.0**	**9.1±3.0**	**0.9±0.0**	10.4±2.2	**36.2±12.1**	1.2±0.1	1.2±0.1	**0.1±0.1**	**0.2±0.0**
		p	**0.001**	**< 0.0001**	**0.019**	**< 0.0001**	**0.001**	0.252	**< 0.0001**	0.241	0.360	**< 0.0001**	**0.0008**
		pre-ex	**0.2±0.1**	**0.4±0.3**	**1.0±0.0**	**53.4±38.0**	**0.9±0.1**	12.8±6.8	**213.0±151.7**	1.2±0.2	1.3±0.2	**0.3±0.2**	**0.3±0.1**
**B**	M	post-ex	**0.1±0.0**	**0.2±0.1**	**1.0±0.0**	**10.0±3.3**	**0.9±0.0**	10.2±2.0	**39.9±13.0**	1.2±0.1	1.2±0.1	**0.1±0.0**	**0.2±0.0**
		p	**< 0.0001**	**< 0.0001**	**0.0006**	**<0.0001**	**< 0.0001**	0.188	**<0.0001**	0.833	0.197	**<0.0001**	**<0.0001**
		pre-ex	**0.2±0.1**	**0.5±0.3**	**1.0±0.0**	**54.9±38.6**	**0.9±0.1**	12.7±6.8	**218.9±154.1**	1.2±0.3	1.3±0.3	**0.4±0.2**	**0.3±0.1**
	H	post-ex	**0.1±0.0**	**0.2±0.1**	**1.0±0.0**	**11.4±5.7**	**0.9±0.0**	10.8±2.1	**45.5±22.8**	1.2±0.1	1.2±0.1	**0.2±0.1**	**0.2±0.0**
		p	**< 0.0001**	**0.0004**	**< 0.0001**	**< 0.0001**	**0.0004**	0.406	**< 0.0001**	0.855	0.208	**< 0.0001**	**0.0002**

R - Red component; G - Green component; B - Blue component. AngScMom - angular second moment/energy; Correlat - correlation; SumOfSqs - sum of squares; InvDefMom - inverse different moment/homogeneity; SumAverg - summation mean; SumVarnc - summation variance; SumEntrp - summation entropy; DifVarnc - differential variance; DifEntrp - differential entropy; p - the level of marginal significance; SD - standard deviation.
